# Biofilm-disrupting heterojunction microneedles: dual ROS amplification and glucose deprivation for accelerated diabetic wound healing

**DOI:** 10.7150/thno.120787

**Published:** 2025-08-30

**Authors:** Wenjie You, Feng Xiao, Zichao Cai, Jiaxin Zhao, Zhengyao Zhang, Weikang Hu, Yun Chen, Kwang Leong Choy, Zijian Wang

**Affiliations:** 1 Orthopaedic Hospital, Postdoctoral Innovation Practice Base, The First Affiliated Hospital, Jiangxi Medical College, Nanchang University, Nanchang, 330006, China.; 2 Department of Urology, Cancer Precision Diagnosis and Treatment and Translational Medicine Hubei Engineering Research Center, Zhongnan Hospital of Wuhan University, Wuhan 430071, China.; 3 Department of Biomedical Engineering, Hubei Province Key Laboratory of Allergy and Immune Related Disease, TaiKang Medical School (School of Basic Medical Sciences), Wuhan University, Wuhan 430071, China.; 4 School of Materials Science and Engineering, Stem Cells and Tissue Engineering Manufacture Center, Hubei University, Wuhan 430062, China.; 5 Division of Natural and Applied Sciences, Duke Kunshan University, Kunshan 215316, China.

**Keywords:** MXene, microneedle, heterojunction, antibacterial, wound healing

## Abstract

**Rationale:** Diabetic wound healing process is critically hindered by bacterial infection, bacterial biofilm formation, and persistent hyperglycemia. Biomolecular microneedles represent a promising alternative to conventional therapies such as antibiotics and antibiotic-loaded wound dressings, owing to the advantages like reduced risk of drug resistance and enhanced long-term efficacy. However, the microneedles that fulfill the clinical needs of diabetic wounds have rarely been reported.

**Methods:** A glucose oxidase (GOx)-laden Ti_3_C_2_/In_2_O_3_ (INTG) heterojunction was engineered as a nano-micro platform for reactive oxygen species (ROS) amplification and glucose deprivation, and subsequently immobilized onto the gelatin methacryloyl (GelMA) microneedle tips to obtain double-layer microneedles (GITG microneedles). Their physiochemical properties and biomedical applications were comprehensively investigated.

**Results:** For INTG heterojunction, the formation of Schottky structure significantly improved the oxygen absorption capacity, facilitated the generation and migration of photogenerated electron-hole pairs, thereby promoting the ROS generation. Besides, under near-infrared (NIR) irradiation, GITG microneedles effectively inhibited bacterial proliferation and survival by generating ROS, thereby preventing the formation of bacterial biofilm. Additionally, GITG microneedles accelerated wound closure and facilitated skin tissue regeneration in a rat model through multiple mechanisms.

**Conclusion:** This study developed an advanced microneedle platform enabling on-demand multimodal treatment, demonstrating significant potential for clinical diabetic wound management.

## Introduction

Diabetic skin wounds (DSWs) constitute one of the main causes of disability and death in individuals with diabetes 1. From 1990 to 2022, the number of adults diagnosed with diabetes (≥ 18 years old) has increased from 200 million to 828 million worldwide 2. DSWs account for approximately one-third of the total diabetes-related healthcare expenditures, imposing a substantial socioeconomic burden 3. The pathogenesis of DSWs includes local hyperglycemia, hypoxia and neurovascular injury, which are often accompanied by bacterial infection, bacterial biofilm formation and the destruction of deeper tissue 4-6. In recent years, many theranostic strategies and techniques have been developed to improve the clinical outcomes of DSWs. Among them, biomolecular biomaterials, such as hydrogels, sponges, electrospun nanofibers and 3D-printed scaffolds, are superior owing to their desirable biocompatibility, biodegradability, well-defined structure and functions 7-9. Biomolecular biomaterials show significant promise for both fundamental research and clinical translation.

Microneedles (MN) are composed of micro-sized tips connected to a base layer 10-12. Several raw materials, such as silk fibroin (SF) and hyaluronic acid (HA), have been applied to fabricate biomolecular MN 13-15. These MNs painlessly puncture the skin with minimal risk of infection, delivering their preloaded cargoes for precise treatment 16-19. In recent years, biomolecular MNs have emerged as an ideal approach for accelerating wound healing 20, 21. Gelatin methacryloyl (GelMA) is a photocurable molecule derived from gelatin 22. Previous studies have reported several GelMA-based MN 23-25. They absorb wound exudates, maintain a clean and moist wound microenvironment (WME), and promote the adhesion and migration of fibroblasts. However, neat GelMA MN lack glucose-depleting, antibacterial and anti-biofilm capabilities, thereby limiting their application potential for diabetic wound management. Thus, it is necessary to modify GelMA MN to endow them with specific bioactivities.

Engineered nanomaterials are immobilized into the tips of GelMA MN, revealing a promising modification strategy 26. Indium oxide (In_2_O_3_) nanoparticles are n-type semiconductors with a wide bandgap and high catalytic activity 27. In_2_O_3_ nanoparticles generate reactive oxygen species (ROS) by photocatalytic therapy, effectively eliminating bacterial infection and inhibiting the formation of bacterial biofilms 28. Glucose oxidase (GOx) catalyzes glucose to produce gluconic acid and hydrogen peroxide (H_2_O_2_) under physiological condition. Thus, it is widely used for glucose deprivation therapy in diabetes 29. MXenes, first reported by Yury Gogotsi et al. in 2011, are a family of 2D transition metal carbides, nitrides, and carbonitrides 30. Monolayer Ti_3_C_2_ nanosheets occupy a central position among MXenes, due to their high specific surface area, abundant functional groups, and excellent conductivity 31. In the pre-experiments, we used Ti_3_C_2_ nanosheets as a micro-nano platform to integrate In_2_O_3_ nanoparticles and GOx. Ti_3_C_2_/In_2_O_3_ heterojunction was synthesized using a hydrothermal method, and then coated with GOx by physical adsorption. The results have demonstrated enhanced ROS amplification and glucose deprivation abilities. However, the mechanism remains to be elucidated.

Heterojunctions combine two or more functional materials with distinct characteristics 32, 33. Owing to the spatial potential difference, the electron-hole pairs at the interface of heterojunction are effectively separated 34, significantly improving the catalytic potential compared with that of single material. Heterojunctions were initially explored as cascaded ROS amplifiers for biomedical applications 35-37. In this study, n-type semiconductor In_2_O_3_ nanoparticles were integrated with Ti_3_C_2_ nanosheets which possess metallic properties. It was supposed to construct a Schottky heterojunction to effectively improve the ROS amplification performance. DFT analysis is an effective method for elucidating the mechanism of heterojunction-mediated catalytic performance 38, 39. This study employed DFT analysis by calculating several key parameters such as the density of states, differential charge density and adsorption energy, enhancing the understanding of charge transfer and photocatalytic mechanisms of Ti_3_C_2_/In_2_O_3_ heterojunction at a theoretical level 40.

**Figure 1:**
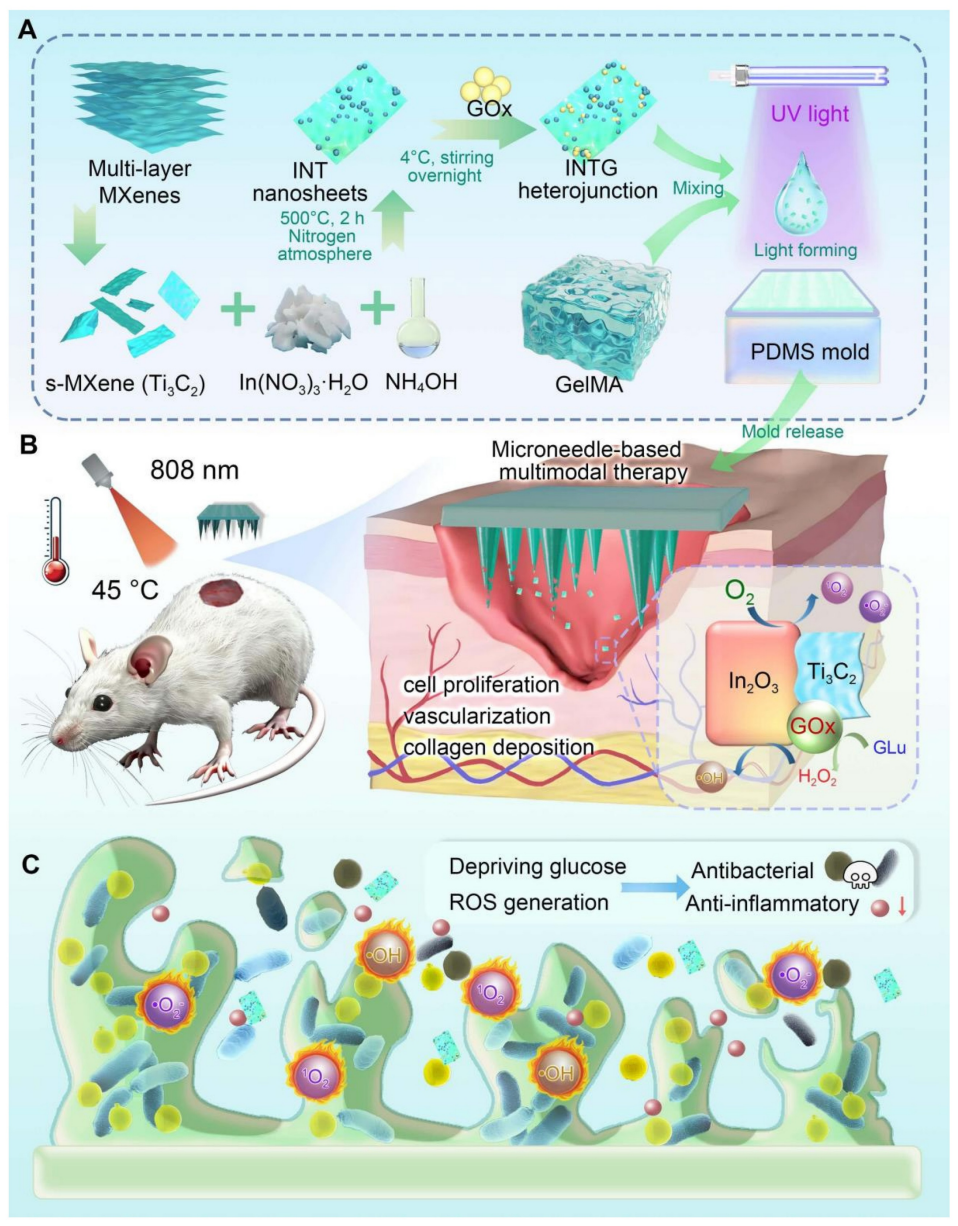
** Preparation of the GOx-laden Ti_3_C_2_/In_2_O_3_ heterojunction composite microneedle platform and its biomedical application.** (A) An INTG heterojunction was prepared and immobilized onto the GelMA-based double-layer microneedles. (B) Composite microneedles combined with mild photothermal therapy accelerate diabetic wound healing via the production of ROS and the deprivation of glucose. (C) A diagram illustrating the antibacterial and antibiofilm mechanisms.

A diagram of this study is shown in **Figure 1**. Monolayer Ti_3_C_2_ nanosheets were chemically etched from bulk MAX. Ti_3_C_2_/In_2_O_3_ heterojunction was synthesized by a hydrothermal reaction and then loaded with GOx. The obtained INTG heterojunction product was subsequently immobilized onto the GelMA MN tip arrays via a mold-casting technique. It is hypothesized that the composite MN produce ROS to eliminate bacterial infection, inhibit the formation of bacterial biofilms, and deplete glucose *in situ*. This study will comprehensively investigate the pro-oxidative mechanism and therapeutic efficacy of this MN in wound healing. The study offers a biocompatible, photo-responsive platform enabling on-demand multimodal treatment, demonstrating potential for treating diabetic wound and others wounds.

## Results and Discussion

### Preparation and characterization of the GOx-laden Ti_3_C_2_/In_2_O_3_ (INTG) heterojunctions

#### Synthesis of INTG heterojunctions

**Figure 2:**
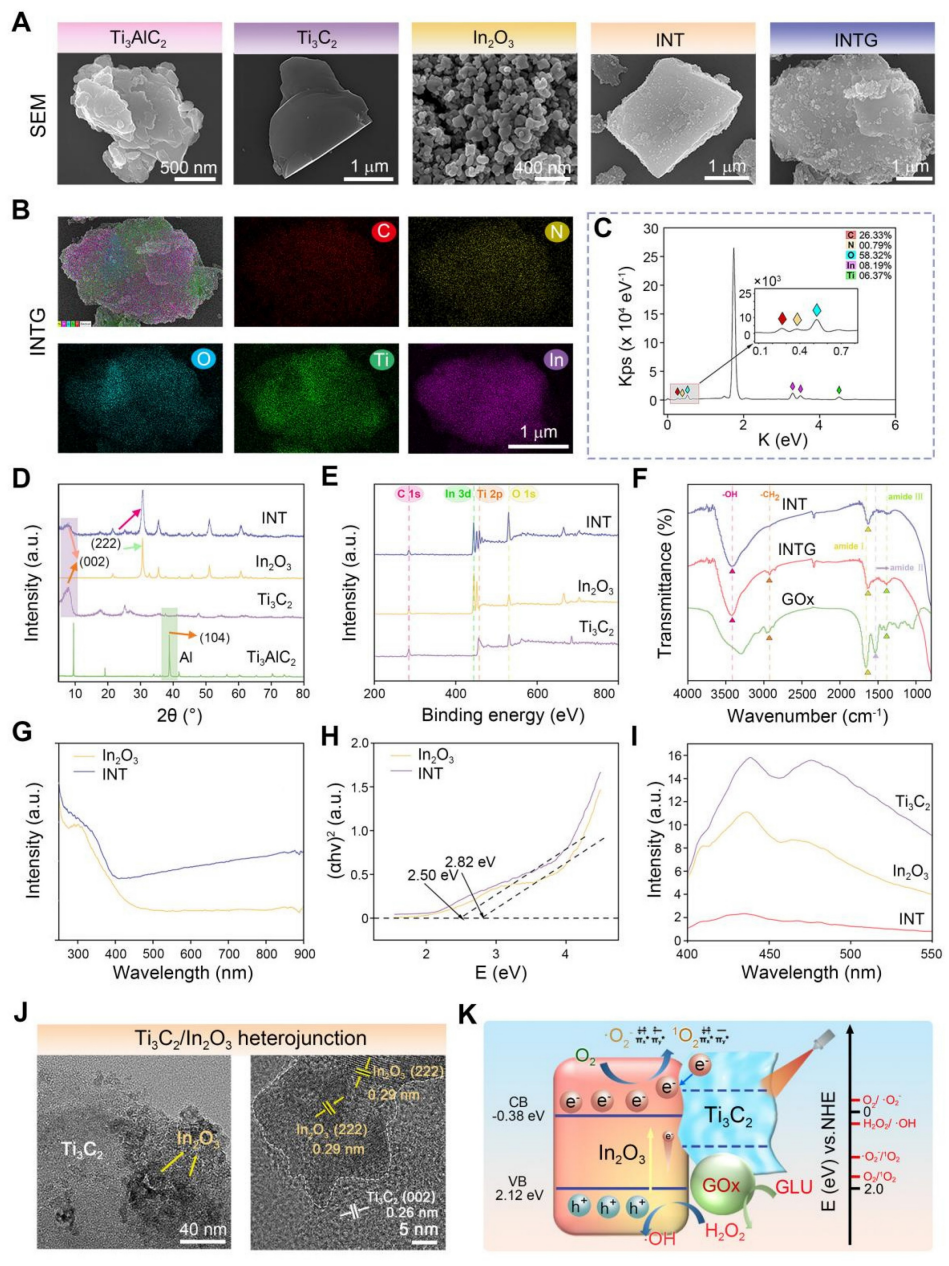
** Characterization of the GOx-laden Ti_3_C_2_/In_2_O_3_ (INTG) heterojunction**. (A) SEM images of bulk Ti_3_AlC_2_, monolayer Ti_3_C_2_ nanosheets, In_2_O_3_ nanoparticles, Ti_3_C_2_/In_2_O_3_ (INT) heterojunction (INT) and INTG heterojunction. Scale bar: 500 nm, 400 nm or 1 μm as indicated. (B-C) SEM image and elemental analysis of the INTG heterojunction, showing the distribution of C, N, O, Ti, and In. Scale bar: 1 μm. (D) XRD spectrum. (E) XPS spectrum. (F) FT-IR spectrum. (G) UV-Vis spectrum. (H) The bandgap energy as calculated using the Tauc-Plot method. (I) PL spectrum. (J) TEM images of an INT heterojunction. Scale bar: 40 nm or 5 nm. (k) Diagram showing the pro-oxidative potential of the INTG heterojunction.

As shown in **Figure 2**A, bulk MAX (Ti_3_AlC_2_) was exfoliated into monolayer MXene (Ti_3_C_2_) nanosheets using an improved hydrofluoric acid etching method. Compared to bulk Ti_3_AlC_2_, the Ti_3_C_2_ nanosheets exhibited a significantly increased specific surface area, making them an ideal nano-micro platform for chemical modifications and biomedical applications. In_2_O_3_ nanoparticles were then synthesized *in situ* onto Ti_3_C_2_ nanosheets using a hydrothermal method, yielding a Ti_3_C_2_/In_2_O_3_ (INT) heterojunction. In_2_O_3_ nanoparticles were evenly distributed on the surface of the INT heterojunction owing to the mild reaction environment. Finally, the INT heterojunction was co-incubated with a GOx solution for physical immobilization. As a result, INTG heterojunction was successfully obtained. SEM mapping was performed to visualize the element components. As shown in **Figure 2**B, INTG heterojunction exhibited the characteristic elements of Ti_3_C_2_ (Ti and C), In_2_O_3_ (In and O), and GOx (N). The quantitative results are presented in **Figure 2**C, showing relative contents of 26.33% C, 0.79% N, 58.32% O, 8.19% In, and 6.37% Ti, respectively.

XRD analysis was performed to characterize the crystallinity of the nanomaterials (**Figure 2**D). Compared with bulk Ti_3_AlC_2_, the characteristic peak (104) of the Al layer disappeared from the Ti_3_C_2_ nanosheets. Moreover, the (002) characteristic peak of Ti_3_C_2_ shifted from 9.6° to 7.1°, which is consistent with the previous report 41. This phenomenon confirmed that the Ti_3_C_2_ nanosheets were successfully exfoliated. In_2_O_3_ nanoparticles exhibited four characteristic peaks located at 30.68° (222), 35.48° (400), 51.10° (440), and 60.73° (622). The characteristic peaks of both Ti_3_C_2_ nanosheets and In_2_O_3_ nanoparticles were found in the INT group. The INT heterojunction simultaneously exhibited characteristic XPS peaks of both Ti_3_C_2_ and In_2_O_3_ (**Figure 2**E). The In3d, O1s, Ti2p and C1s XPS spectra of INT heterojunction are shown in **Figure S1**. These results confirmed the successful synthesis of INT heterojunction comprising Ti_3_C_2_ nanosheets and In_2_O_3_ nanoparticles. **Figure 2**F shows the FT-IR spectrum. The characteristic peaks of neat GOx were located at 1659 cm^-1^ (amide I band), 1540 cm^-1^ (amide II band), 1231 cm^-1^ (amide III band), 1393 cm^-1^ (deformation vibration of the CH_2_ group), 2964 cm^-1^ (stretching vibration of the C-H group), and 3298 cm^-1^ (stretching vibration of the hydroxyl group). The characteristic peaks of the complex (INTG group) located at 3417 cm^-1^ (stretching vibration of the hydroxyl O-H), 2924 cm^-1^ (stretching vibration of the C-H group), 1631 cm^-1^ (amide I band) and 1389 cm^-1^ (deformation vibration of CH₂) were characteristic peaks of GOx. These results revealed the interaction between the INT heterojunction and GOx protein. This interaction altered the structure of GOx, which in turn influenced the wavenumbers of the infrared absorption peaks.

#### INTG heterojunctions exhibit pro-oxidative potential

The light absorption performance of heterojunction plays a crucial role in their photocatalytic and photothermal properties.** Figure 2**G shows the ultraviolet (UV)-visible diffuse reflectance spectrum. In_2_O_3_ nanoparticles exhibited strong light absorption in the wavelength range of 250 - 400 nm, while the INT heterojunction demonstrated broad and intense light absorption across 250 - 900 nm. In particular, in the near-infrared (NIR) range of 780 - 900 nm, INT heterojunction exhibited stronger light absorption ability than In_2_O_3_ nanoparticles. UV-visible diffuse reflectance data were used to calculate the bandgap energy of heterojunction (**Figure 2**H). The bandgap energy of In_2_O_3_ nanoparticles was measured at 2.82 eV (vs. NHE), whereas the bandgap of INT heterojunction exhibited a reduced bandgap of 2.50 eV (vs. NHE). The reduced bandgap energy suggested the enhanced electron excitation into the conduction band, facilitating easier electron transitions from the valence band to conduction band. To determine the electron-hole recombination rate of the nanomaterial, photoluminescence (PL) spectroscopy was performed (**Figure 2**I). The electron-hole recombination rate of INT heterojunction was significantly lower than that of In_2_O_3_ nanoparticles and the Ti_3_C_2_ nanosheets. These findings indicated that a greater number of photogenerated electrons were involved in the catalytic reactions and photothermal conversion processes. This phenomenon can be attributed to the effective design and modification of heterojunction structure, which significantly improved the photocatalytic performance and photothermal conversion ability of the composite heterojunction. TEM analysis showed that the interplanar spacing of In_2_O_3_ in the INT heterojunction was 0.29 nm and that the interplanar spacing of Ti_3_C_2_ was 0.26 nm (**Figure 2**J). These results further confirmed the successful construction of heterojunction structure.

In this study, the incorporation of GOx into the INTG heterojunction was expected to eliminate glucose and transform it into oxidative H_2_O_2_ in diabetic patients 42, and the incorporation of INT heterojunction was expected to endow the INTG heterojunction with increased pro-oxidative potential. By fitting the bandgap data (**Figure 2**H) with XPS valence band spectra (**Figure S2**A-B), the band structure diagram of the INT heterojunction (**Figure 2**K) was obtained. The conduction band value of the INT heterojunction was -0.38 eV (vs. NHE), and the valence band value was 2.12 eV (vs. NHE). A comparison of the standard oxidation-reduction potentials for generating ROS revealed that the photogenerated electrons produced by the INTG heterojunction effectively converted the dissolved oxygen in water into •O_2_^-^ radicals, whereas the electron-hole pairs converted H_2_O_2_ generated by GOx into more oxidatively active •OH.

### Verification of the pro-oxidative effect and mechanism of the INTG heterojunction

**Figure 3:**
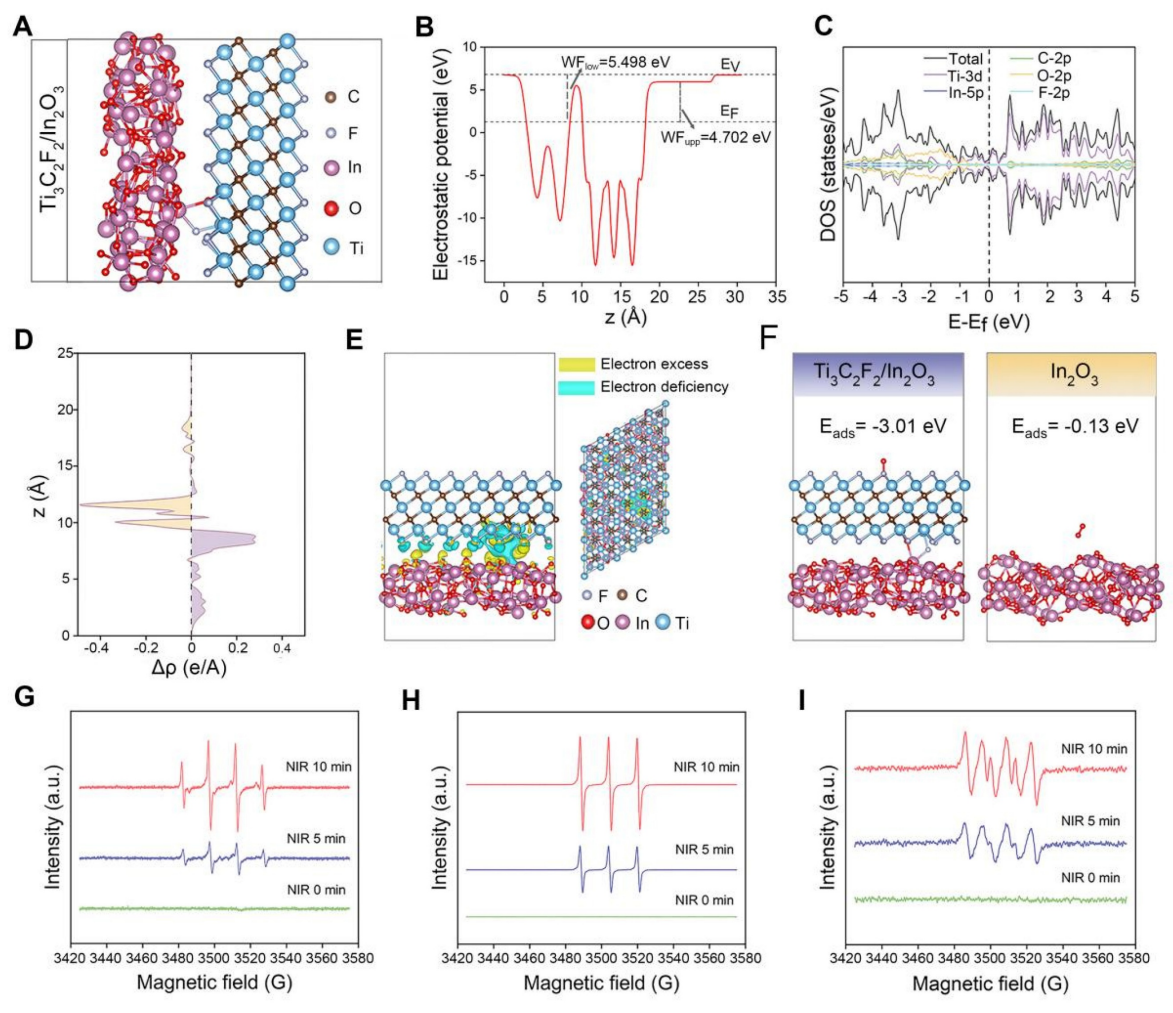
** Verification of the pro-oxidative effect and mechanism of the INTG heterojunction by DFT calculations**. (A) DFT calculation model of In_2_O_3_/Ti_3_C_2_F_2_. (B) Work function of the In_2_O_3_/Ti_3_C_2_F_2_ interfaces where E_V_ represents the vacuum level; E_F_ represents the Fermi level; “upp” represents the upper surface; and “low” represents the lower surface of INTG heterojunction. (C) Projected density of state (DOS). (D) Differential charge densities of the In_2_O_3_/Ti_3_C_2_F_2_ interfaces and the planar-averaged differential charge density ∆ρ along the Z direction. (E) Differential charge density model of In_2_O_3_/Ti_3_C_2_F_2_. The yellow area represents the region of electron excess, and the cyan area represents the region of electron deficiency. (F) Oxygen adsorption model of In_2_O_3_/Ti_3_C_2_F_2_ and In_2_O_3_ nanoparticles. The pink, red, blue, brown, and silver/white colors correspond to indium (In), oxygen (O), titanium (Ti), carbon (C), and fluorine (F) atoms, respectively. (G) EPR spectra of ·OH. (H) EPR spectra of ^1^O_2_. (i) EPR spectra of ·O_2_^-^.

The pro-oxidative effect of the INTG heterojunction is attributed to the incorporation of the INT heterojunction. In the present study, a density functional theory (DFT) calculation was performed to reveal the pro-oxidative mechanism, with a focus on the generation and transfer of electrons. The constructed theoretical model is shown in **Figure 3**A. The surface of the In_2_O_3_ nanoparticles was constructed with 120 atoms, and the cells were expanded. Ti_3_C_2_ nanosheets with a total of 364 atoms were capped with F atoms to construct the heterojunction. The formation of F vacancies exposed metal adsorption sites, enabling O_2_ adsorption. As shown in **Figure 3**B, In_2_O_3_ nanoparticles and Ti_3_C_2_ nanoparticles formed a Schottky heterojunction, as Ti_3_C_2_ exhibited metallic properties, consistent with previous reports 43. The work function of the lower surface (WF_low_) of the INT heterojunction was 5.498 eV, and the work function of the upper surface (WF_upp_) of the INT heterojunction was 4.702 eV.

To further explore the structural optimization, the total and partial density of states (DOS) at the interfaces of the INT heterojunction were systematically calculated (**Figure 3**C). The curve of the total DOS crossed through the Fermi level, indicating that the INT heterojunction exhibited metallic properties. The results of the partial DOS indicated that the metallic properties were derived from the Ti_3_C_2_ nanosheets. In addition, on the two sides of the Fermi level, the distribution of the total DOS was attributed mainly to O-2p and Ti-3d. Next, a differential charge density analysis was performed (**Figure 3**D-E). An area of electron deficiency was observed in the Ti_3_C_2_ layer, whereas an area of electron excess was observed in the In_2_O_3_ layer. This phenomenon indicated that the electrons were transferred from the Ti_3_C_2_ nanosheets to the In_2_O_3_ nanoparticles, resulting in a charge density distribution along the Z-axis direction. Overall, these results revealed that the structure of the INT heterojunction facilitated the generation of photogenerated electrons, thererby theoretically enhancing the production of oxidative ROS. Notably, the results of the DFT calculations were highly consistent with the results shown in **Figure 2**G-I.

An oxygen adsorption model was constructed for further verification. As shown in **Figure 3**F, the energy of oxygen adsorption (E_ads_) of the INT heterojunction was -3.01 eV, whereas the E_ads_ of the In_2_O_3_ nanoparticles was -0.13 eV. These findings indicated that the formation of the INT heterojunction improved the oxygen adsorption ability. The ROS-regenerating ability of the INT heterojunction under NIR irradiation was detected by electron paramagnetic resonance (EPR). As shown in **Figure 3**G-I, the characteristic peaks of ·OH with a ratio of 1:2:2:1, ^1^O_2_ with a ratio of 1:1:1, and ·O_2_^-^ with four major peaks and two minor peaks were clearly observed. The intensity of these peaks increased with longer NIR exposure time. In summary, these results indicated that the INT heterojunction combined with NIR irradiation would generate oxidative ROS. The potential mechanism is attributed to the formation of a heterojunction structure.

### Preparation of INTG-laden GelMA hydrogels and double-layer MNs

#### Characterization of INTG-laden GelMA hydrogels

**Figure 4:**
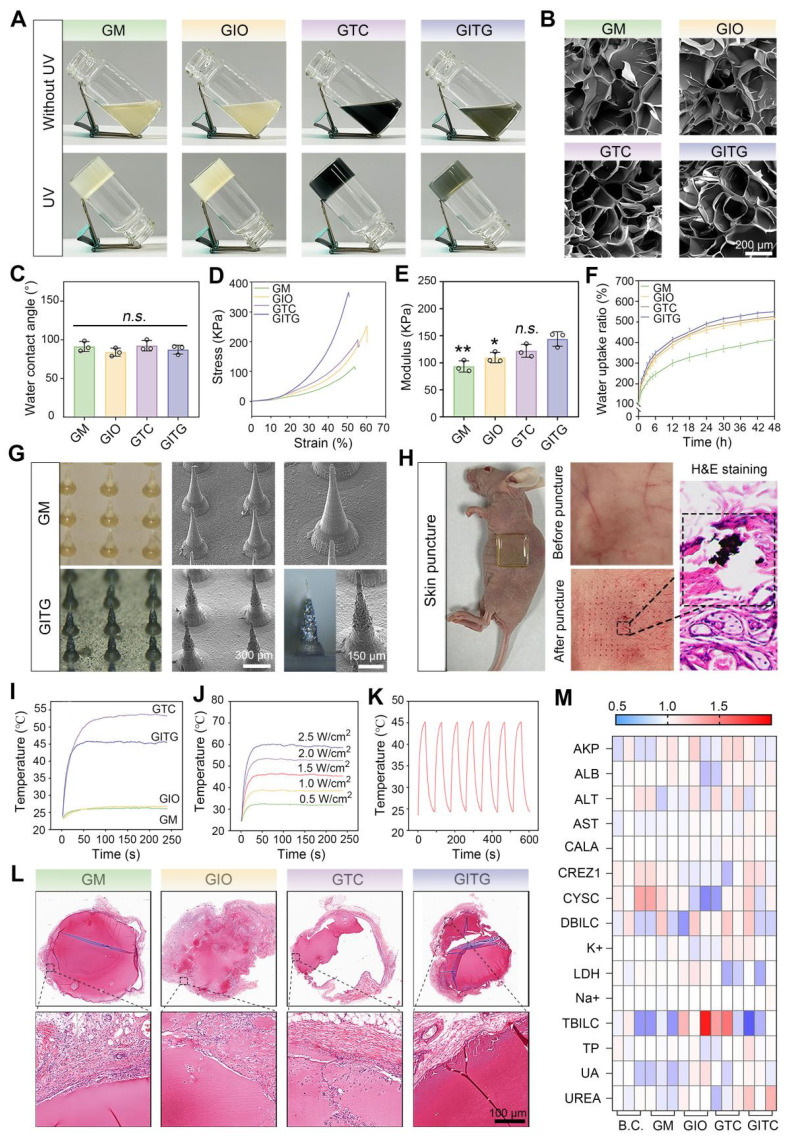
** Characterization of INTG-laden double-layer GelMA hydrogels and microneedles.** (A) Optical images of GelMA hydrogels (GM), GelMA/In_2_O_3_ hydrogels (GIO), GelMA/Ti_2_C_3_ hydrogels (GTC) and GelMA/INTG hydrogels (GITG) before and after UV crosslinking. (B) SEM images of freeze-dried hydrogels. Scale bar: 200 μm. (C) Water contact angles (n = 3) of GM, GIO, GTC and GITG. (D-E) Compressive test results (n = 3) of different hydrogels. (F) Dynamics of the water uptake ratio (n = 3) of various hydrogels. (G) Morphological observation of double-layer GM and GITG microneedles. Scale bar: 300 μm or 150 μm. (H) The GITG microneedles punctured the skin of BALB/c nude mice. (I) Photothermal curves of different microneedles. (J) Photothermal curves of GITG microneedles at different NIR powers. (K) Cyclic photothermal curves of GITG microneedles. (L) H&E staining images of neofibrous capsules. Scale bar: 100 μm. (M) Heatmap of blood biochemical tests (n = 3) of a blank control and various hydrogels. The values are expressed as the mean ± standard deviation (SD). Compared with the GITG group, *n.s.* indicates no significance, *P < 0.05, and **P < 0.01.

In this study, four types of hydrogels (GM, GIO, GTC and GITG) were prepared using ultraviolet (UV)-mediated crosslinking 44. As illustrated in **Figure 4**A, the solution transitioned from a liquid state to a solid-state after UV irradiation at a power of 300 W for 300 s. GelMA molecules and photoinitiators (2959) cross-linked to form a supramolecular network, serving as a mechanical-tough skeleton of composite materials 45. The nanomaterials, such as In_2_O_3_ nanoparticles, Ti_3_C_2_ nanosheets and INTG heterojunctions, were physically immobilized into the supramolecular network. No additional chemical reactions occurred, thereby avoiding potential side effects to humans. As shown in **Figure 4**B, the interior of the hydrogels exhibited a typical porous structure. The cavities formed during freeze‒drying were beneficial for absorbing wound exudates and mechanically adapting to irregular wound shapes.

The physical properties of the hydrogels were comprehensively characterized. The hydrophilicity was assessed via water contact angle measurements (**Figure 4**C and **Figure S3**), revealing no significant differences among the four groups (Define P, *P* > 0.05). A compressive test was performed to evaluate the mechanical strength of the hydrogels (**Figure 4**D-E). The elastic modulus was 93.3 ± 10.4 KPa for the GM hydrogel, 109.7 ± 9.6 KPa for the GIO hydrogel, 122.2 ± 11.9 KPa for the GTC hydrogel, and 144.1 ± 13.4 KPa for the GITG hydrogel. Significant differences were observed when the GITG group was compared with the other groups. As shown in **Figure 4**F, the hydrogels absorbed a large amount of water within the first 12 h, indicating good swelling ability. Compared with the GM hydrogel, the incorporation of nanomaterials significantly enhanced the swelling ability.

#### Characterization of INTG-laden double-layer MNs

As in our previous report 46, the hydrogels were processed into MN using a mold-casting technique. In this study, a double-layered structure with a base layer of GM hydrogel and a tip layer of composite hydrogel was designed. The base layer primarily provides mechanical support, while the tip layer would puncture the skin and deliver bioactive nanomaterials for treatment 47. This double-layered structure not only reduces the consumption of expensive nanomaterials but also minimizes potential side effects associated with their use. Morphological observation of the double-layer MN revealed that the GM MN tips were composed of a GM hydrogel and that the GITG MN tips were composed of a GITG hydrogel (**Figure 4**G and **Figure S4)**. The tips of the GITG MN were much darker and rougher than those of GM MN due to the incorporation of the INTG heterojunction. The external points of the tips were sharp. As shown in **Figure 4**H, the GITG MN punctured the skin of BALB/c nude mice, indicating that the mechanical strength and sharpness of the tips are sufficient to fulfill clinical needs. Four types of double-layer microneedles, specifically GM MN, GIO MN, GTCMN and GITG MN were obtained and characterized in this study. Both GTC and GITG MN demonstrated superior photothermal conversion efficiency compared to the other groups (**Figure 4**I, **Figure S5**), suggesting strong potential for photothermal therapy (PTT) applications. Previous studies 48-51 have demonstrated that PTT can effectively eradicate bacterial infection and bacterial biofilms regulating macrophage phenotype and improving blood supply.

Thus, PTT is useful in terms of accelerating diabetic wound healing. In this study, the photothermal effect of GITG MN was effectively regulated by the NIR light (**Figure 4**J). Furthermore, the GITG MN maintained excellent photothermal stability over at least seven heating‒cooling cycles (**Figure 4**K).

#### Biocompatibility evaluations of INTG-laden double-layer MN

The biocompatibility of the microneedles was evaluated using a subcutaneous transplantation model in Sprague-Dawley (SD) rats. As shown in **Figure 4**L, the microneedles were transplanted *in vivo* for 14 days, and neocapsule tissue surrounded the microneedles. The capsule did not have a dense tissue structure, and there was no massive immune cell infiltration. A series of immunofluorescence (IF) staining assays were performed to evaluate the inflammation stage. As shown in **Figure S6**A-B, the relative protein expression of IL-6 and CD45 did not significantly differ among the four groups (*P* > 0.05), indicating that these microneedles would not trigger obvious immunological rejection 52, 53.

Blood and organ samples from the treated SD rats were also collected for a series of biocompatibility tests. Blood biochemical tests indicated that the levels of each indicator were within the normal range (**Figure 4**M). Compared with the blank control (BC) group, each indicator was not significantly different (*P* > 0.05, **Figure S7**), suggesting good hemocompatibility. H&E staining revealed no obvious pathological changes in the brain, heart, liver, spleen, lung and kidney among the five groups, suggesting good tissue compatibility (**Figure S8**). Collectively, these findings demonstrated that the microneedles exhibited good biocompatibility and met the standards for Class III medical devices.

### GITG MN have pro-oxidative nanozyme-like activity under NIR irradiation

**Figure 5:**
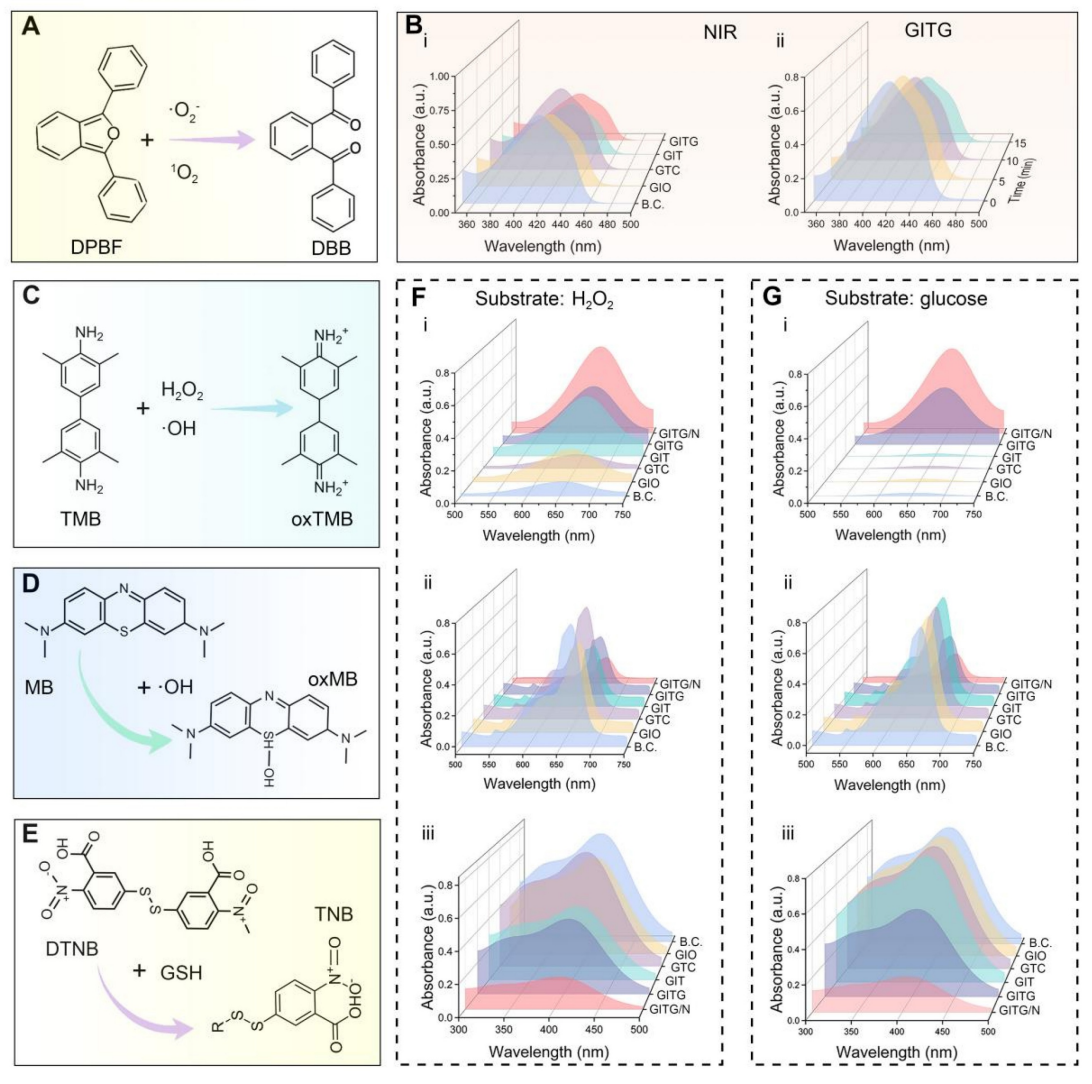
** Pro-oxidative evaluation of NIR-assisted GITG microneedles.** (A) Diagram showing the reaction formula of the DPBF assay. (B) Results of the DPBF assay. (C-E) Diagrams showing the reaction formulas of the TMB assay, MB assay and DTNB assay. (F) Corresponding results using H_2_O_2_ as a substrate. (G) Corresponding results using glucose as a substrate.

The above results demonstrated the pro-oxidative properties of the INTG heterojunction. Owing to its lower oxygen adsorption energy (-3.01 eV) and heterojunction structure, the INTG heterojunction can generate oxidative ·OH, ^1^O_2_ and ·O_2_^-^ under NIR irradiation. To determine if the incorporation of the INTG heterojunction would endow GITG MN with enhanced pro-oxidative properties, biochemical assays were performed. **Figure 5**A shows the reaction formula of the 1,3-diphenylisobenzofuran (DPBF) assay. DPBF was oxidized to o-dibenzoyl benzene (DBB) by ^1^O_2_ and·O_2_^-^, resulting in solution fading and a decrease in the absorbance value at 410 nm. As shown in **Figure 5**B(i), both the GITG and GIT MNs exhibited a decrease in absorbance under NIR irradiation. Moreover, the absorbance of GITG MN would gradually decrease with longer exposure time (**Figure 5**B (ii)). These results indicated that GITG MN would produce ^1^O_2_ and ·O_2_^-^ by the photodynamic treatment.

As shown in **Figure 5**C, 3,3',5,5'-tetramethylbenzidine (TMB) is oxidized to ox-TMB by H_2_O_2_ and ·OH. The results of TMB assay using a substrate of H_2_O_2_ or glucose are shown in **Figure 5**F(i) and** Figure 5**G(i), respectively. The GITG/N group showed increased absorbance at 652 nm. The quantitative results are shown in **Figure S10**A (i) and **Figure S10**B (i), and significant differences were observed between the GITG/N group and the other groups (*P* < 0.001). These results were attributed to POD-like nanozyme activity. A methylene blue (MB) assay was used to detect the ability of each group to produce ·OH (**Figure 5**D). Regardless of whether the substrate was H_2_O_2_ or glucose, the GITG/N group presented the lowest absorbance at 664 nm, followed by the GITG group (**Figure 5**F(ii), **Figure 5**G(ii)). The quantitative results are shown in **Figure S10**A (ii) and **Figure S10**B (ii), and significant differences were observed between the GITG/N group and the other groups (*P* < 0.001). Without glucose, there was no discrepancy among the BC, GIO, GTC and GIT MN groups. These results indicated that GITG MN not only could produce H_2_O_2_ from glucose by GOx but also generate ·OH through POD-like enzyme activity and photodynamic performance.

Glutathione (GSH) is the most important antioxidant against oxidative stress in organisms, including bacteria. To evaluate GSH oxidation capacity, we performed the 5,5'-dithiobis-2-nitrobenzoic acid (DTNB) assay, the common method for GSH quantification (**Figure 5**E). In the presence of H_2_O_2_, absorbance at 410 nm decreased in GIO, GIT, GTC and GITG/N MN groups (**Figure 5**F(iii)). However, the effect of the GIO group was weaker, and groups containing INT heterojunctions consumed more GSH. Under glucose conditions (**Figure 5**G(iii)), the GITG group and GITG/N group still consumed a large amount of GSH due to GOx activity. With NIR irradiation, the temperature and long-wavelength photodynamic performance increased GSH consumption in the GITG/N group. The quantitative data are shown in **Figure S10**A(iii) and **Figure S10**B(iii). These findings indicated that when there is enough glucose in the environment, GITG MN would disrupt the antioxidant system and kill bacteria.

### GITG MN capable of eliminating bacterial infection and bacterial biofilms

#### Evaluation of a broad-spectrum antibacterial activity

**Figure 6:**
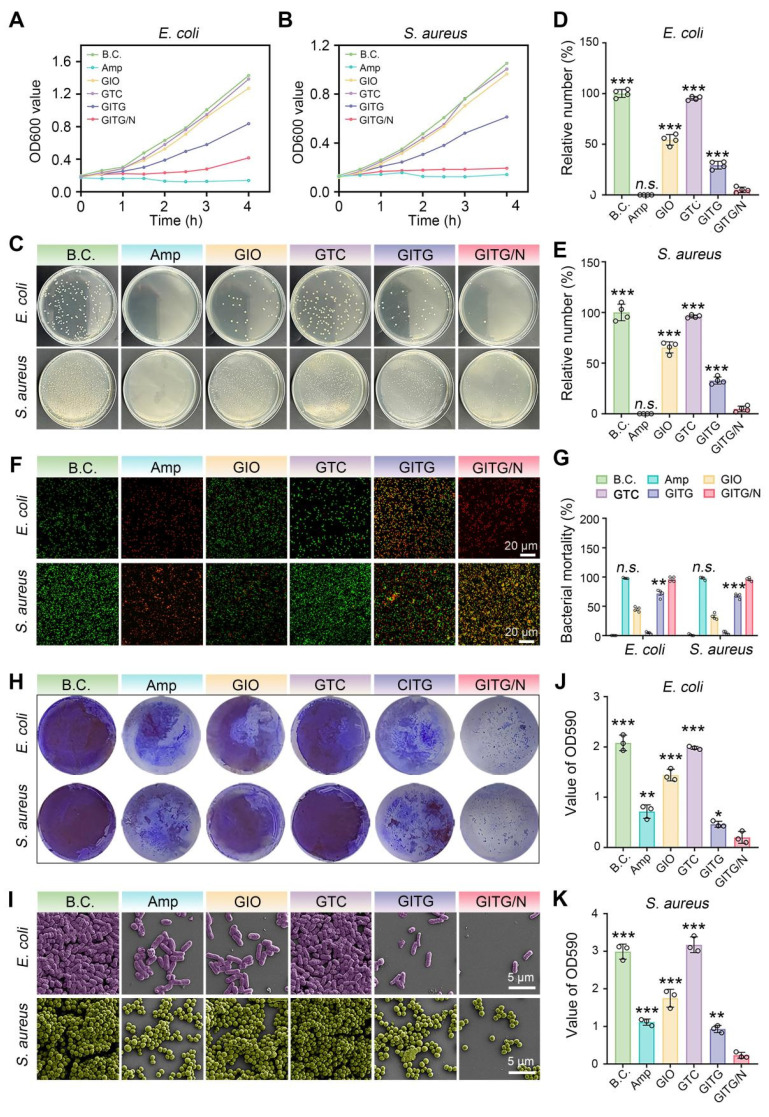
** Evaluation of broad-spectrum antibacterial and antibiofilm activities of NIR-assisted GITG microneedles.** (A) Proliferation curves of *E. coli*. (B) Proliferation curves of *S. aureus*. (C) Optical images of bacterial clones. (D) Corresponding results for *E. coli* (n = 4). (E) Corresponding results for *S. aureus* (n = 4). (F) Live/dead bacteria staining images. Scale bar: 20 μm. (G) Quantitative results of bacterial mortality (n = 4). (H) Crystal violet staining of bacterial biofilms. (I) SEM images of bacterial biofilms. Scale bar: 5 μm. (J) Corresponding results for *E. coli* of crystal violet staining (n = 3). (K) Corresponding results for *S. aureus* of crystal violet staining (n = 3). The values are expressed as the mean ± SD. Compared with the GITG/N group, *n.s.* indicates no significance, *P < 0.05, **P < 0.01, and ***P < 0.001.

GITG MN combined with PTT (GITG/N) can generate a large amount of ROS, representing a promising approach for antibacterial and antibiofilm treatment 54. In this study, the broad-spectrum antibacterial and antibiofilm activities of GITG MN combined with and without mild PTT were comprehensively investigated using two kinds of model organisms, namely, gram-negative* E. coli* bacteria and gram-positive* S. aureus* bacteria 55. The positive control group was treated with a sensitive antibiotic (ampicillin, Amp). The results of the bacterial proliferation assay are shown in **Figure 6**A-B. GITG/N and Amp effectively inhibited the proliferation of both *E. coli* and *S. aureus*, revealing good antibacterial activity. The antibacterial effect of the GITG/N group was significantly better than that of the GITG group. This phenomenon suggested that the antibacterial effect was attributed mainly to the ROS generated by GITG/N rather than by the GITG MN themselves. Meanwhile, the results of the bacterial ROS staining were showed in **Figure S9**. The fluorescence intensity of ROS in GIO group and GITG group was brighter than that in B.C. group and Amp group, which could benefit from the POD-like nanozyme activity of In_2_O_3_. And the fluorescence intensity of ROS in GITG/N group was the brightest, which indicated GITG/N killed bacteria by generating ROS.

The bacterial survival ability was evaluated via a colony formation assay (**Figure 6**C). For both the* E. coli* and *S. aureus* bacteria, the Amp and GITG/N groups presented fewer bacterial clones than the other groups. The quantitative results are shown in **Figure 6**D-E. For *E. coli* bacteria, the relative number of bacterial clones was 100 ± 3.92% for the BC group, 0 ± 0% for the Amp group, 54.31 ± 5.22% for the GIO group, 95.28 ± 1.83% for the GTC group, 29.33 ± 3.80% for the GTIG group and 5.05 ± 2.69% for the GITG/N group. For *S. aureus* bacteria, the relative number of bacterial clones was 100 ± 8.20% for the BC group, 0 ± 0% for the Amp group, 65.48 ± 5.47% for the GIO group, 96.03 ± 1.46% for the GTC group, 32.8 ± 3.53% for the GTIG group and 4.78 ± 2.80% for the GITG/N group. No significant difference was observed between the Amp and GTIG/N groups (P > 0.05), suggesting that the anti-survival ability of GITG/N treatment was comparable to that of sensitive antibiotics. As shown in** Figure 6**F, a live/dead bacteria staining assay was performed to visualize bacterial viability. Live bacteria were dyed green, while dead bacteria were stained green and red, which merged into yellow. **Figure 6**G showed the bacterial mortality calculated from the live/dead bacteria staining assay. The Amp and GITG/N groups presented the greatest bacterial mortality, which is consistent with the previous results shown in **Figure 6**A-E.

#### Evaluation of antibacterial and antibiofilm activities

Bacterial biofilms are a primary cause of recurrent infections in diabetic patients. Live bacteria produce extracellular polymeric substances (EPSs) and adhere to the surface of the substrate, forming a drug resistance barrier 56, 57. To maintain the integrity of biofilms, bacterial adhesion mediated by proteins and other factors plays a vital role, particularly in bacterial communication and interactions. In this study, the antibiofilm activity of the GITG/N treatment was investigated. A crystal violet staining assay was performed to quantitatively measure the bacterial biofilm. As shown in **Figure 6**H and J-K, fewer bacterial biofilms were found in the GITG/N group. For *E. coli*, the OD590 value was 2.08 ± 0.15 for the BC group, 0.71 ± 0.13 for the Amp group, 1.44 ± 0.12 for the GIO group, 1.98 ± 0.03 for the GTC group, 0.46 ± 0.06 for the GTIG group and 0.20 ± 0.12 for the GITG/N group. For *S. aureus*, the OD590 value was 2.99 ± 0.20 for the BC group, 1.11 ± 0.08 for the Amp group, 1.75 ± 0.24 for the GIO group, 3.17 ± 0.21 for the GTC group, 0.93 ± 0.10 for the GTIG group and 0.23 ± 0.07 for the GITG/N group. Compared with the GITG/N group, there were significant differences in the OD590 values in the other groups (*P* < 0.05). Besides, the SEM results are shown in** Figure 6**I. For the BC group, many bacteria adhered to the glass sheet, resulting in dense bacterial biofilms. For the Amp and GITG/N groups, fewer bacteria adhered to the glass sheets, resulting in sparse bacterial biofilms after treatment, but no morphological changes of bacteria were observed in the SEM results.

Bacterial autoaggregation is one of the key factors contributing to the shaping and maturation of biofilm communities and extracellular polymeric substances (EPS) is another key factor to form biofilms, maintain the stability of biofilm skeleton and protect bacteria in biofilms from the damage of antibacterial drugs. Therefore, the bacterial autoaggregation experiment and EPS detection experiment were conducted. **Figure S11**A-B showed GITG/N treatment significantly reduced the autoaggregation, only 9.07 ± 1.35 % for *E. coli* and 15.13 ± 3.07 % for *S. aureus* compared with the autoaggregation of B.C. group, 56.06 ± 4.08 % for *E. coli* and 87.78 ± 3.47 % for *S. aureus*. Furthermore, the results of EPS were showed in the **Figure S12**A-B. Compared with the other groups, the polysaccharide and protein concentration on biofilms of GITG/N group were significantly reduced. The results indicated that GITG/N treatment can inhibit the secretion of polysaccharides and proteins in the biofilm matrix, further impacting the initial adhesion process of bacteria and leading to a delay or absence in the formation of mature biofilms.

These results suggested that GITG/N treatment has effectively inhibited the proliferation, survival and bacterial biofilm formation of both gram-positive and gram-negative bacteria. The broad-spectrum antibacterial and antibiofilm activities of GITG/N treatment are attributed mainly to the burst of ROS generated by the GITG MN. Moreover, the incorporation of mild PTT might also increase the sensitivity of bacteria to ROS 58. The GITG/N treatment developed in this study represents a promising antibiotic alternative with broad-spectrum antibacterial applications, particularly for infected wound healing.

### GITG MN accelerate diabetic wound healing

**Figure 7:**
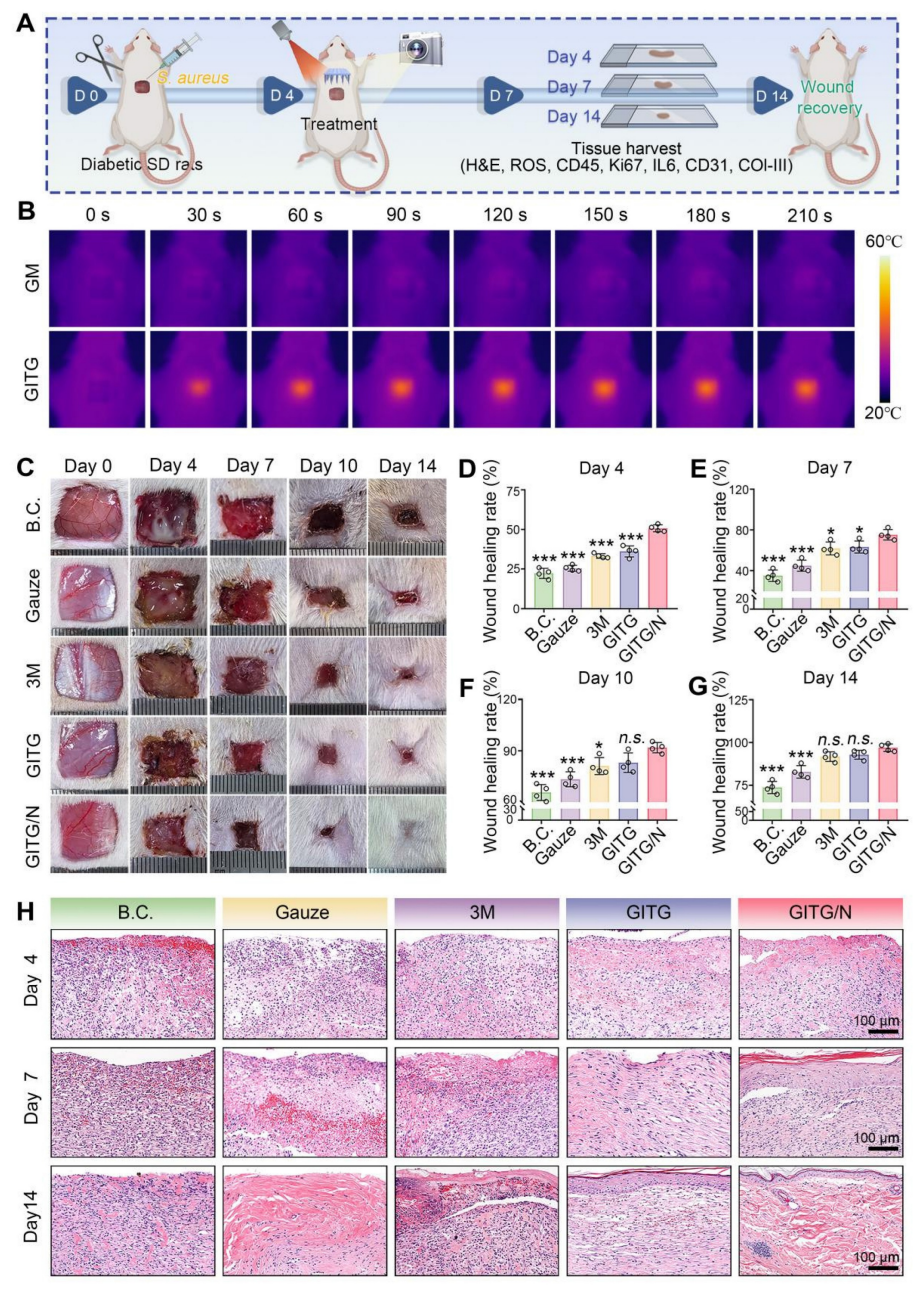
***In Vivo* evaluation of wound-healing*.*** (A) Diagram showing the experimental protocols. (B) Photothermal treatment *in vivo*. (C) Optical images of the wound sites. (D-G) Quantitative results of the wound-healing rate (n = 4). (H) Representative images of H&E staining of neo-skin tissues. Scale bar: 100 μm. Values are expressed as the mean ± SD. Compared with the GITG/N group, *n.s.* indicates no significance, *P < 0.05, **P < 0.01, and ***P < 0.001.

An *S. aureus*-infected diabetic skin defect model in SD rats was constructed for application evaluation* in vivo* (**Figure 7**A). Diabetes was successfully induced in these animals via streptozotocin (STZ) administration (**Figure S13**) 59. The wounds were covered with a piece of GITG MN and then treated with or without NIR irradiation (GITG group and GITG/N group) several times. The blank control group was left untreated. The negative control group was treated with medical gauze, and the positive control group was treated with an antibacterial 3M wound dressing. After surgery, all the animals were conventionally fed in a specific pathogen-free (SPF) environment to allow skin tissue regeneration. As shown in **Figure 7**B, GITG MN exhibited better *in vivo* photothermal properties than GM MN, and these properties satisfied the general requirements for *in vivo* animal-based evaluations.

The wounds almost healed within 14 days, as shown in photographs of the wound sites (**Figure 7**C). The wound-healing rates at four time points were quantitatively measured (**Figure 7**D-G). The GITG/N group healed the fastest among the five groups, and significant differences were observed between the GITG/N group and the other groups (*P* < 0.05). Neo-skin tissue was resected for H&E staining and analysis (**Figure 7**H). On Day 4, fewer inflammatory cells infiltrated the GITG/N group, suggesting good antibacterial effects and biocompatibility. On Day 7, a large amount of granulation tissue filled the skin defects in the five groups. The GITG/N group presented keratinizing epithelium, whereas keratinizing epithelium was not observed in the other groups. On Day 14, complete keratinizing epithelium, hair follicles, and dense collagen fibers were observed in the GITG/N group but not in the other groups. These results confirmed that the GITG/N treatment has effectively accelerated wound closure and promoted skin tissue regeneration.

**Figure 8:**
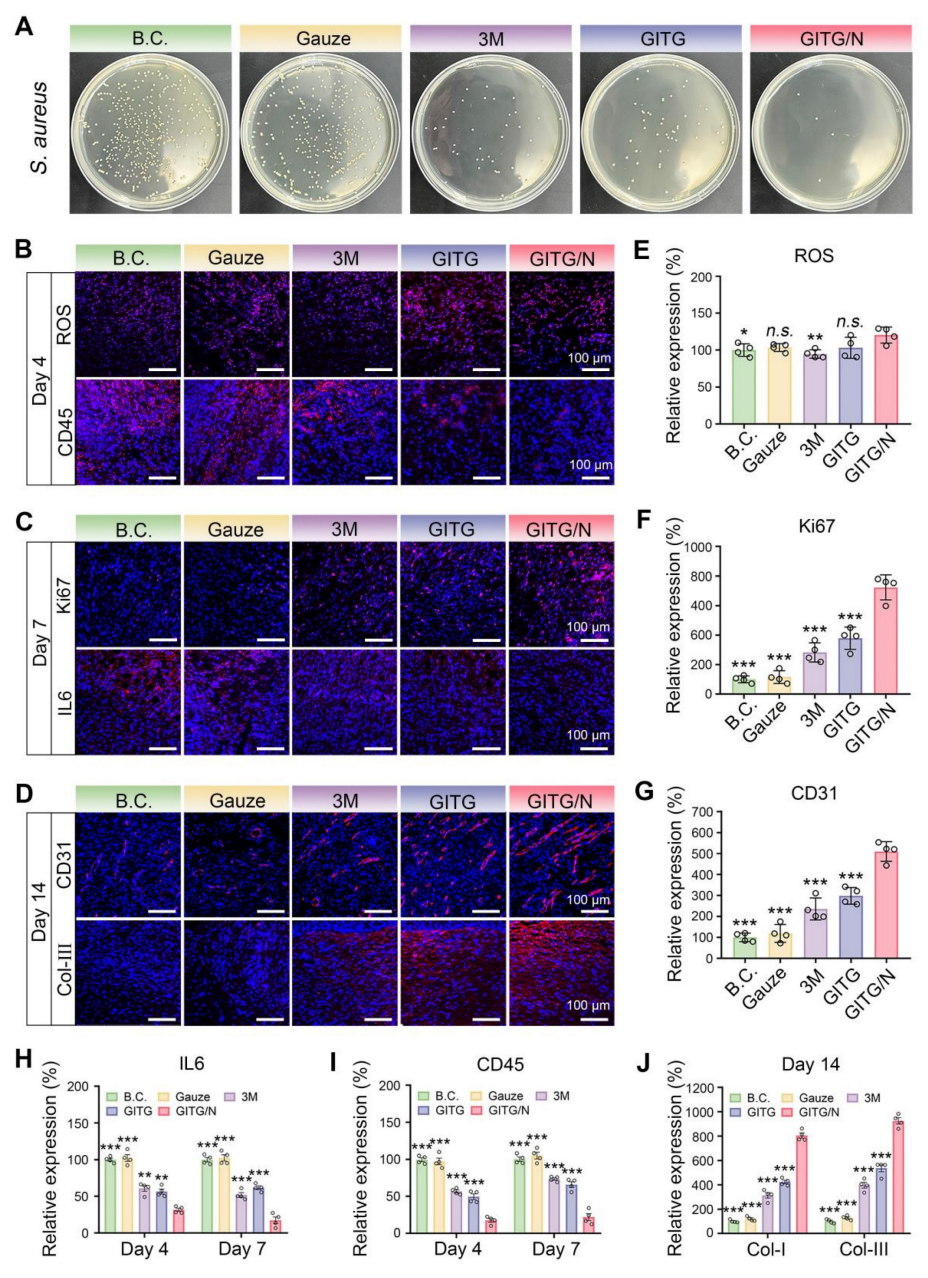
** Mechanism for accelerated wound healing.** (A) *In vivo* antibacterial evaluation. (B) IF staining images of ROS and CD45 on Day 4. Scale bar: 100 μm. (C) IF staining images of IL6 and Ki67 on Day 7. Scale bar: 100 μm. (D) IF staining images of Col-III and CD31 on Day 14. Scale bar: 100 μm. (E) Relative expression of ROS on Day 4 (n = 4). (F) Relative expression of Ki67 on Day 7 (n = 4). (G) Relative expression of CD31 on Day 14 (n = 4). (H-J) Relative expression of IL6, CD45 and Col-I and Col-III (n = 4). The values are expressed as the mean ± SD. Compared with the GITG/N group, *n.s.* indicates no significance, *P < 0.05, **P < 0.01, and ***P < 0.001.

The antibacterial effects of the different materials were verified* in vivo* (**Figure 8**A and **Figure S14**). The relative number of bacterial clones was 100 ± 9.49% for the BC group, 98.11 ± 13.04% for the gauze group, 19.40 ± 4.42% for the 3M group, 20.22 ± 6.92% for the GITG group and 3.40 ± 3.66% for the GITG/N group. The GITG/N group exhibited the best antibacterial effect, owing mainly to the burst of ROS and photothermal effects generated by GITG/N treatment. In the early stages of wound healing, the antibacterial effect is one of the hub factors, as it inhibits bacterial metabolism, reduces the inflammatory response, and promotes tissue repair by improving the harsh wound microenvironment 60, 61.

Neo-skin tissue was resected for a series of IF staining analyses. On Day 4, markers of oxidative stress (ROS) and inflammatory intensity (IL6 and CD45) were detected (**Figure 8**B and **Figure S16**C). Compared with those in the other groups, the expression of ROS was slightly elevated in the GITG/N group, but the expression levels of IL-6 and CD45 were significantly decreased (*P* < 0.001). On Day 7, Ki67 (marker of proliferation), CD45 and IL6 were detected (**Figure 8**C and **Figure S16**B). Compared with the other groups, the GITG/N group presented the highest expression levels of Ki67 but the lowest expression levels of IL-6 and CD45 (*P* < 0.001). On Day 14, vascularization (CD31) and collagen remodeling (Col-I and Col-III) markers were detected (**Figure 8**D and **Figure S16**C). Compared with the other groups, the GITG/N group presented the highest expression levels of CD31, Col-I and Col-III (*P* < 0.001). The quantitative results and significant differences of each indicator are shown in **Figure 8**E-J.

In summary, the GITG/N treatment has accelerated diabetic wound healing via antibacterial and anti-inflammatory activities, thereby improving cell proliferation, vascularization, collagen deposition and remodeling. However, the GITG/N treatment showed limited effects on wound ROS levels. In this study, ROS were only produced under NIR irradiation and quickly reacted with bacteria or were eliminated. The GITG/N group typically showed no detectable ROS production without NIR irradiation. The GTIG/N group also depleted glucose via GOx, which inhibited bacterial survival and proliferation, thereby alleviating oxidative stress injury and maintaining good biocompatibility.

## Conclusions

This study developed double-layer GITG MN with broad-spectrum antibacterial and antifilm properties to enhance diabetic wound healing, while maintaining excellent biocompatibility. An INTG heterojunction was immobilized onto the GITG MN tip arrays for transdermal delivery, functioning as a nano-micro platform to amplify ROS and deplete glucose. The INTG heterojunction structure enhanced the oxygen absorption ability, promoted the transfer of photogenerated electrons, and increased the production of ROS. The combination of GITG MN with mild PTT (GITG/N) has effectively inhibited bacterial proliferation and survival, and prevented the formation of bacterial biofilms by producing a burst of ROS. The overall antibacterial effect of GITG/N treatment was comparable to that of sensitive antibiotics, such as ampicillin. An *S. aureus*-infected diabetic skin defect model in SD rats was constructed to evaluate the effects of GITG/N treatment* in vivo*. The GITG/N treatment significantly accelerated wound closure and promoted skin tissue regeneration by depleting glucose, enhancing antibacterial and anti-inflammatory activities and improving cell proliferation, vascularization, collagen deposition and remodeling. The regenerative efficacy of the GITG/N treatment surpassed that of antibacterial 3M wound dressings. Thus, these findings demonstrate that the GITG MN developed in this study have great clinical potential for diabetic wound management.

## Methods

### Materials

Indium nitrate hydrate (In(NO_3_)_3_·xH_2_O), lithium chloride (LiCl), titanium aluminum carbide (Ti_3_AlC_2_), 5,5-dimethyl-1-pyrroline N-oxide (DMPO), methacrylic anhydride (MA), 2,2,6,6-tetramethyl-4-piperidone hydrochloride (TEMP), and ammonia solution were purchased from Aladdin Co., Ltd. (Shanghai, China). GOx and gelatin were obtained from Macklin Biotech. Co., Ltd. (Shanghai, China). The microneedle mold was provided by Zhongding Yuxuan Co., Ltd. (Hefei, China). *Escherichia coli* (*E. coli*) and *Staphylococcus aureus* (*S. aureus*) were obtained from the China General Microbiological Culture Collection Center. All chemicals and reagents were used as-received.

### Preparation and characterization of INTG heterojunctions

Monolayer Ti_3_C_2_ nanosheets were etched from bulk Ti_3_AlC_2_. In brief, 1 g of Ti_3_AlC_2_ was added to hydrofluoric acid and reacted at 35 ℃ for 12 h. The supernatant was separated via centrifugation at 3500 rpm for 5 min. The residual precipitate was washed three times with distilled water. The precipitate (0.05 g/mL) was added to the LiCl solution and allowed to react for 12 h.

Finally, the above mixture was sonicated for 1 h and centrifuged to obtain the Ti_3_C_2_ nanosheets. 30 mL of absolute ethanol and 7 mL of ammonia (25%) were mixed, and 100 mg of Ti_3_C_2_ was added to the mixture. Then, 100 mg of In(NO_3_)_3_·xH_2_O was added to the solution. The solution was transferred to a reactor and reacted at 120 ℃ for 24 h, and subsequently centrifuged at 5000 rpm for 5 min to obtain a gray solid powder. The solid powder was calcined at 500 °C for 2 h in a tubular furnace under nitrogen protection to obtain Ti_3_C_2_/In_2_O_3_ (INT). Add a sentence on how you prepare the INT solution (i.e. INT powder in what kind of solvent etc). Is this solution or INT being suspended in a solvent. The INT (10 mg/mL) solution was sonicated for 30 min, and an equal volume of GOx (20 µg/mL) was added into the INT solution, followed by stirring at 4 ℃ overnight. Finally, the resultant solution was freeze-dried to obtain GOx-laden Ti_3_C_2_/In_2_O_3_ (INTG) heterojunctions. The synthesis method of In_2_O_3_ was consistent with the previous report 62.

A scanning electron microscope (Zeiss Gemini 300) was used to observe the micromorphology of the materials, and an energy dispersive spectrometer was used for element detection. X-ray diffraction (XRD, X'Pert PRO MPD), Fourier transform infrared spectroscopy (FTIR, Nexus 670) and X-ray photoelectron spectroscopy (XPS, Thermo ESCALAB 250XI) were used to determine the material composition. The heterojunction structure of INTG was verified via UV-visible absorption spectroscopy (PE lambda 750), valence band X-ray diffraction (VB-XPS, X'Pert PRO MPD), photoluminescence spectroscopy (PL, FLS980 Series of Fluorescence Spectrometers) and transmission electron microscopy (TEM, FEI Talos F200X).

### Verification of the pro-oxidative effect and mechanism

The structural model of the INT heterojunction was constructed based on first principles calculations. Details of the DFT calculation are provided in the supporting information (SI). To detect hydroxyl radicals (·OH), 160 µL of INTG (5 mg/mL) and 20 μL of DMPO were mixed, and the changes in electron paramagnetic resonance (EPR) spectra over time under NIR irradiation were recorded. The detection of superoxide anion (·O_2_^-^) and tertiary butyl alcohol was used to eliminate ·OH, and the other details were the same as those used for the detection of ·OH. Singlet oxygen (^1^O_2_) was detected using TEMP as a capture agent. A total of 160 µL of INTG (5 mg/mL) and 20 μL of TEMP were mixed, and the changes in EPR were recorded over time under NIR irradiation.

### Preparation and characterization of INTG-laden GelMA hydrogels and double-layer microneedles

GelMA was obtained by crosslinking gelatin and MA, according to our previously reported methods. In brief, 2 g of GelMA was dissolved in 8 mL of water to prepare a 20 wt% GelMA solution. Next, 20 mg of INTG was added to the mixture, and a solution of INTG-laden GelMA was obtained after magnetic stirring for 30 min and centrifugation. The obtained solution was poured into the corresponding mold and cross-linked under UV light (500 W) for 5 min, yielding a hydrogel named GITG. For comparison, GelMA hydrogel, GelMA/Ti_3_C_2_ hydrogel, GelMA/In_2_O_3_ hydrogel and GelMA/INT were prepared using the same method and named GM, GTC, GIO, and GIT, respectively.

The gel-forming process of the hydrogels was recorded using a phone camera, and the morphology of the hydrogels was observed by SEM. A compressive test was performed with an electronic universal testing machine (CMT6103), and the water contact angle was measured using a contact angle tester (Dataphysics OCA20). To evaluate the swelling capacity and the time required to reach swelling equilibrium, freeze-dried hydrogels from each group were first weighed and then immersed in saline solution. The mass was recorded at different time points, and the water uptake ratio was calculated. Double-layer microneedles of GITG were synthesized according to the following three steps. Firstly, to obtain the needle tip layer, the INTG-laden GelMA solution was poured into the mold, and air bubbles were removed using a vacuum drying oven. The solution containing bubbles in the upper layer was carefully removed using a cotton swab. Secondly, a 20 wt% GelMA solution was added to the upper layer of the mold and gelled using UV light (500 W) for 5 min. Finally, the double-layer microneedles were prepared after drying and demolding.

The other experimental methods are provided in the supporting information.

### Statistical analysis

Quantitative data are expressed as mean ± standard deviation (SD). For comparisons between two independent groups, independent-sample t-tests were employed, while multiple group comparisons were analyzed using one-way analysis of variance (ANOVA) followed by Tukey's honestly significant difference (HSD) post hoc test when significant main effects were detected. All statistical analyses were conducted using SPSS software version 21.0 (IBM Corporation, Armonk, NY, USA). Statistical significance was defined as *P < 0.05, **P < 0.01, and ***P < 0.001, and data represent results from at least three independent experiments unless otherwise specified.

## Supplementary Material

Supplementary methods and figures.
