# Supplementary material for: Biofilm-disrupting heterojunction microneedles: dual ROS amplification and glucose deprivation for accelerated diabetic wound healing
Source: Theranostics. 2025 Aug 30;15(18):9757–74. doi: 10.7150/thno.120787 (PMC12486409; doi:10.7150/thno.120787)
Supplement: Supplementary file 1 — Supplementary methods and figures. [file thnov15p9757s1.pdf]

Supplementary information

Biofilm-disrupting heterojunction microneedles: dual ROS amplification and glucose deprivation for accelerated diabetic wound healing

Wenjie You[a,b,1], Feng Xiao[b,1], Zichao Cai[a], Jiaxin Zhao[a], Zhengyao Zhang[b,c], Weikang Hu[d], Yun Chen[c,*], Kwang Leong Choy[e,*] and Zijian Wang[b,c,*]

[a] Orthopaedic Hospital, Postdoctoral Innovation Practice Base, The First Affiliated Hospital, Jiangxi Medical College, Nanchang University, Nanchang, 330006, China

[b] Department of Urology, Cancer Precision Diagnosis and Treatment and Translational Medicine Hubei Engineering Research Center, Zhongnan Hospital of Wuhan

[c] Department of Biomedical Engineering, Hubei Province Key Laboratory of Allergy and Immune Related Disease, TaiKang Medical School (School of Basic Medical Sciences), Wuhan University, Wuhan 430071, China.

[d] School of Materials Science and Engineering, Stem Cells and Tissue Engineering Manufacture Center, Hubei University, Wuhan 430062, China

[e] Division of Natural and Applied Sciences, Duke Kunshan University, Kunshan 215316, China

*

*Corresponding authors at:
Department of Urology, Zhongnan Hospital of Wuhan University, Wuhan 430071, China. E-mail address: Zijianwang@whu.edu.cn (Z. Wang); Division of Natural and Applied Sciences, Duke Kunshan University, Kunshan 215316, China. E-mail address: kwang.choy@duke.edu (K.L.C.); Department of Biomedical Engineering, TaiKang Medical School (School of Basic Medical Sciences), Wuhan University, Wuhan 430071, China. E-mail address: Yunchen@whu.edu.cn (Y.C.).

[1] These authors contributed equally to this work.

Experimental Section

Density functional theory (DFT) calculations

We have employed the first-principles tool -Vienna Ab-initio Simulation Package (VASP) to perform all DFT calculations within the generalized gradient approximation (GGA) using the Perdew-Burke-Ernzerhof (PBE) formulation. We have chosen the projected augmented wave (PAW) potentials to describe the ionic cores and take valence electrons into account using a plane wave basis set with a kinetic energy cutoff of 450 eV. Partial occupancies of the Kohn−Sham orbitals were allowed using the Gaussian smearing method and a width of 0.05 eV. For the optimization of both geometry and lattice size, the Brillouin zone integration was performed with 1×1×1 Γ-centered k-point sampling. The self-consistent calculations applied a convergence energy threshold of 10-5 eV. The equilibrium geometries and lattice constants were optimized with maximum stress on each atom within 0.02 eV Å-1. The 15 Å vacuum layer was normally added to the surface to eliminate the artificial interactions between periodic images. The weak interaction was described by DFT+D3 method using empirical correction in Grimme's scheme. Spin polarization method was adopted to describe the magnetic system. Furthermore, the input files and output data of charge difference and DOS were generated by the tool-Vaspkit. The adsorption energy was calculated as: Eads = E(*adsorbent) - E(*) - E(adsorbent). E(*adsorbent), E(*) and E(adsorbent) represent the total energy of * adsorbent, * and adsorbent molecule, respectively.

Photothermal evaluations

The GM, GIO, GTC, GITG microneedles were irradiated with NIR light with a power of 1.5 W/cm^2 for 4 min. At regular time intervals, the thermal images were photographed by an infrared camera (223s, Fortric, China). The changes of temperature were then recorded. Then GITG microneedles were irradiated with NIR with varied power of 0.5, 1.0, 1.5, 2.0 and 2.5 W/cm^2. The temperature changes were photographed. For cyclic photothermal test, GITG microneedles were irradiated with 1.5 W/cm^2 NIR light. The NIR light was turned on for 40 s, then turned off for 50 s, and repeated for 7

cycles. The changes of temperature were then recorded at regular time intervals.

Biocompatibility evaluations

A subcutaneous transplantation model of SD rats was established for *in vitro* biocompatibility evaluations. Fifteen female SD rats weighing about 200 g were randomly divided into five groups (n = 3). GM, GTC, GIO and GITG groups were transplanted with GM hydrogel, GTC hydrogel, GIO hydrogel and GITG hydrogel, respectively. The blank control (B.C.) group was sham-operated. All animals were conventionally fed for another 14 days. After that, fresh whole blood was collected for a series of blood biochemical tests, which were performed with the help of the Clinical Laboratory of Zhongnan Hospital. The hydrogels, surrounding capsule tissues, and the organs (brain, heart, liver, spleen, lung, and kidney) were also collected for histological analysis.

Pro-oxidative nanozyme-like activities of GITG microneedles assisted by NIR

DPBF assay was performed to detect singlet oxygen. The DPBF solution was obtained by dissolving 1 mg DPBF in 1 mL DMSO. The GIO, GTC, GIT, and GITG microneedles were added to 1 mL DMSO solution and as the control group, the group of no microneedles were named the B.C. group. 10 μL e DPBF solution was subsequently added to the above solution and the solution was given an irradiation of NIR for 30 min. Finally, UV-Vis was used to detect the absorbance intensity of the solution at 410 nm.

TMB assays were performed to detect hydroxyl radical and H_2O_2. 2.4 mg TMB was dissolved in 10 mL absolute ethanol to obtain TMB solution. 1 mL citrate buffer (pH 4.6), the 100 μL TMB solution and 100 μL 2mM H_2O_2 were added to the same test tube. The GIO, GTC, GIT, and GITG microneedles were added to the above tube. The group with no microneedles was named as the B.C. group. The above test tubes were reacted at room temperature for 30 min, while another group of GITG microneedles was irradiated with NIR for 30 min and designated as the GITG/N group. Finally, UV-Vis was used to detect the absorbance intensity of the solution at 652 nm.

MB assays were performed to detect hydroxyl radical. 5 mg MB was dissolved in 2 mL deionized water to obtain MB solution. The reaction solution was prepared by

mixing the 20 μL MB solution and 1mL 2mM H_2O_2. The experimental details were the same as those of TMB assay except the absorbance intensity was detected at 665nm.

DTNB assay was performed to detect the GSH-consuming ability. 4 mg DTNB was dissolved in 10 mL deionized water to obtain DTNB solution. 3 mg GSH was dissolved in 10 mL deionized water to obtain GSH solution. The reaction solution was prepared by mixing the 100 μL DTNB solution, the 100 μL GSH solution, 1 mL citrate buffer (pH 8) and 10 μL 2mM H_2O_2. The experimental details were the same as those of TMB assay except the absorbance intensity detected at 412 nm.

For detecting the GOx ability, the above three experiments were conducted again with a substitution of 2 mM H_2O_2 with 5 wt% glucose solution.

Evaluations of broad-spectrum antibacterial activity

In this study, both gram-negative *E. coli* and gram-positive *S. aureus* served as the model organisms for broad-spectrum antibacterial evaluations. *E. coli* (CICC 10389) and *S. aureus* (CICC 21600) were obtained from China Center of Industrial Culture Collection. The bacteria strain was activated using Luria-Bertani (LB) solid culture medium, and then expanded using LB liquid culture medium. The proliferative bacteria were collected by centrifugation, re-suspended in 0.85% normal saline to inhibit bacterial proliferation, and then incubated with GIO, GTC and GITG microneedles for 4 h. For the GITG/N group, the bacteria were additionally treated with NIR irradiation with a temperature of 45°C for 5 min. The blank control (B.C.) group was not treated, and the positive control (Amp.) group was treated with antibiotics (ampicillin or vancomycin).

Bacterial proliferation assay: 10 μL of bacterial suspension was added to 5 mL of LB liquid medium and shaken at 37 °C. Every 30 min, 3 μL of bacterial culture was sampled, and the value of OD600 absorbance was measured using a microvolume spectrophotometer (Nano-Drop 2000, Thermo, USA). This process was repeated every 30 min for 4 h, resulting in a bacterial growth curve.

Clone formation assay: 100 μL of bacterial suspension was serially diluted 10^2-10^6 times using 0.85% normal saline. Then, 50 μL of each diluted bacterial culture was evenly spread onto LB agar plates and incubated upside down overnight. Digital

photographs of the bacterial clones were taken, and the quantity of bacterial clones was counted.

Live/dead bacteria staining assay: According to the manufacturer's protocols, the treated bacteria were staining using a live/dead bacteria staining kit. 4 μL of bacterial sample was dropped onto a glass slide, and immediately observed using a laser confocal microscope. The percentage of bacterial mortality was calculated using a ImageJ software.

Evaluations of inhibiting bacterial biofilm formation

The bacterial suspension was seeded onto the upper surface of glass slides, and then conventionally cultured for at least 3 days to allow the formation of bacterial biofilm. The amounts of bacterial biofilm were then measured using SEM observation and crystal violet staining assay.

Bacterial autoaggregation experiment: 5 mL LB medium was added to the bacterial suspension after treatment and the suspension was incubated for 6 hours. 1 mL of bacterial suspension with an OD600 of 0.2 and 3 mL of sterile PBS buffer were added to a 5 mL test tube. After vortexed for 10 s, 200 μL of the bacterial suspension from a point 2 cm below the liquid surface was aspirated into a 96-well plate. Then the absorbance A_1 at 600 nm was measured using a microplate reader. The test tubes were incubated in a 37°C water bath for 6 hours. At last, the absorbance A_2 was measured and the bacterial autoaggregation rate was calculated and statistically analyzed.

Bacterial autoaggregation rate = $(A_1 - A_2)/ A_1 * 100\%$.

EPS (Extracellular Polymeric Substances) detection experiment： The determination of polysaccharides and proteins was conducted using the phenol-sulfuric acid method and the Coomassie Brilliant Blue method, respectively. 5 mL of bacterial suspension with an OD600 of 0.2 was seeded onto the upper surface of glass slides, and then conventionally cultured in culture dish for at least 3 days to allow the formation of bacterial biofilm. The biofilm on glass was dissolved in 5 mL a 2% (w/w) EDTA solution containing 2% (w/w) NaCl, and subjected to shaking extraction for 12 hours at 37°C. Once the biofilm was fully dissolved, it was centrifuged at 4°C and 4000 r/min for 30 minutes. The supernatant was then collected for the determination of

polysaccharide and protein content. 1mL of bacterial suspension from culture dish culturing biofilm was added to the centrifuge tube and centrifuged at 10000 r/min for 10 minutes. Subsequently, the same method was used to determine the concentrations of polysaccharides and proteins in the extracellular solution.

Diabetic wound healing test

This study was carried out with the approval of the Animal Welfare and Ethics Committee of the First Affiliated Hospital of Nanchang University (CDYFY-IACUC-202404QR015). Thirty female Sprague-Dawley (SD) rats weighing about 200 g each were kindly provided by the Biotechnology Research Center of China Three Gorges University. All rats were conventionally fed in a specific pathogen-free (SPF) environment to minimize any external stress. According to a previous report, a diabetic model of SD rats was successfully established by streptozotocin (STZ) injection.

All animals were randomly divided into 5 groups (n = 6). Before surgery, they were safely anesthetized by isoflurane inhalation, and their back hair was removed. After disinfecting with a 75% alcohol solution three times, a square full-thickness skin defect was created on each rat and then inoculated with S. aureus suspension. The negative control group was not treated, and the positive control groups were treated with medical gauze and a 3M wound dressing (Tegaderm, USA). The GITG group was treated with GITG microneedles, and the GITG/N group was treated with GITG/N microneedles and photothermal therapy at the same time.

All animals were conventionally fed for another 14 days to allow skin regeneration. At each time point, the wounds were photographed, and the wound area was measured. On day 4, wound exudate was collected for bacterial culture. On day 4, neo-skin tissues were also collected for a series of histological analyses, including H&E staining, immunofluorescence staining of ROS, CD45, IL-6. On day 7, neo-skin tissues were collected for a series of histological analyses, including H&E staining, immunofluorescence staining of CD45, IL-6. CD45, and Ki67. On day 14, neo-skin tissues were collected for a series of histological analyses, including H&E staining, immunofluorescence staining of CD31, Col-I and Col-III. According to standard protocols, histological analysis was performed with the help of Servicebio

Biotechnology Co., Ltd. (Wuhan, China). The images were captured using a laser confocal microscope (Stellaris 5, Leica, Germany).

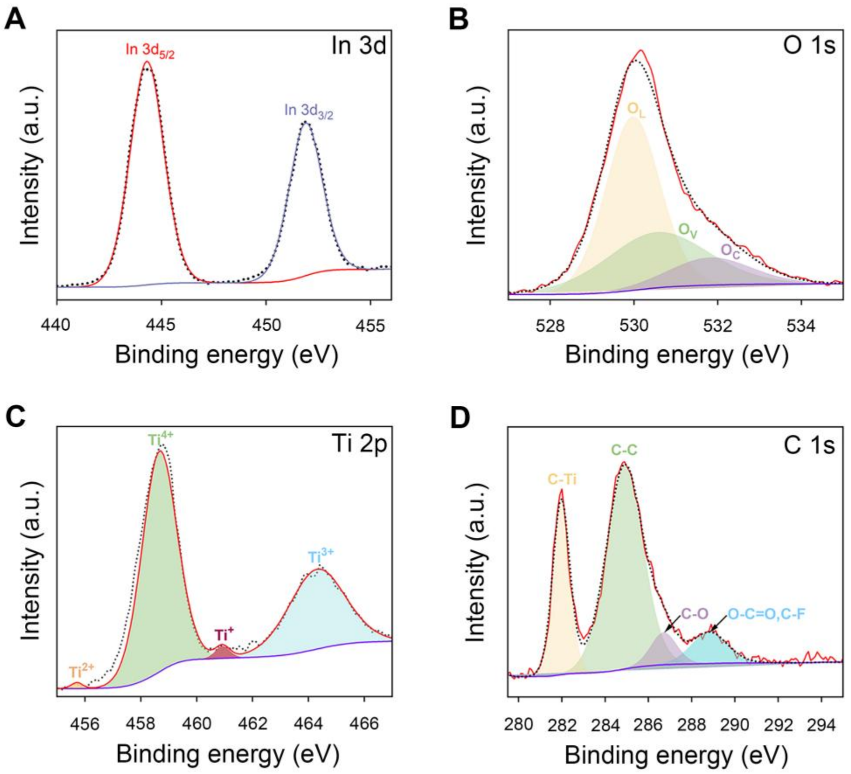

Figure S1. XPS spectrum of INT heterojunction. (A) In 3d XPS spectrum; (B) O 1s XPS spectrum; (C) Ti2p XPS spectrum; and (D) C 1s XPS spectrum.

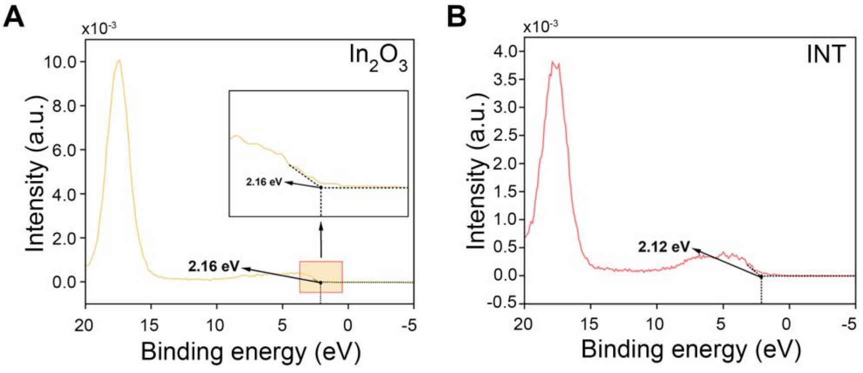

Figure S2. XPS-VB spectrum. (A) XPS-VB spectrum of In_2O_3 nanoparticles; (B) XPS-VB spectrum of INT heterojunction.

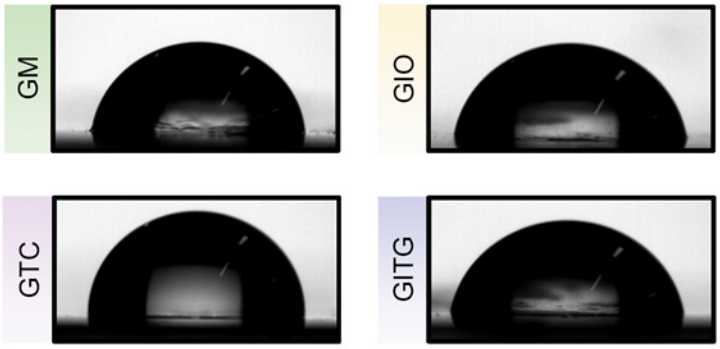

Figure S3. Optical images of water contact angles on the surface of four kinds of hydrogels.

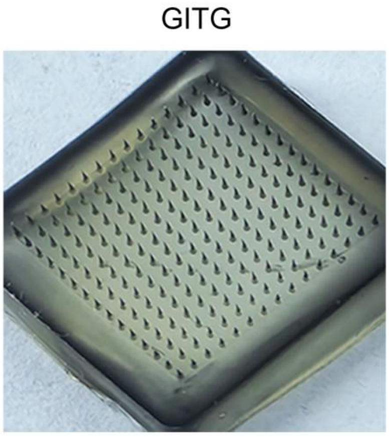

Figure S4. Optical image of double layer GITG microneedles.

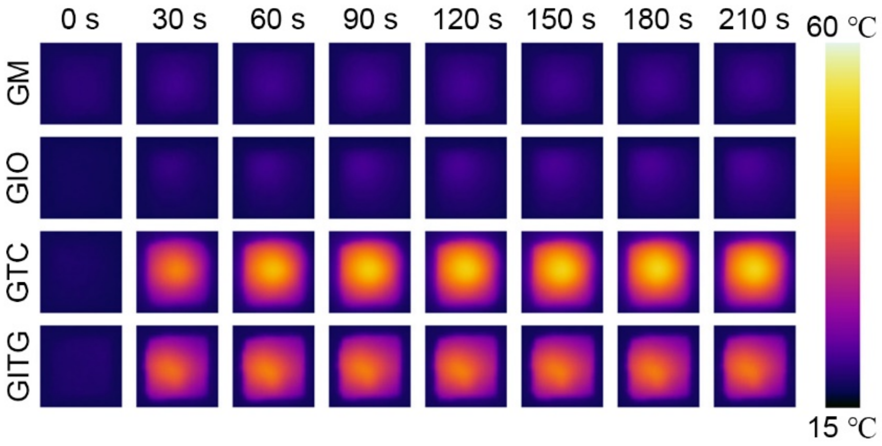

Figure S5. Dynamic photothermal images of four kinds of microneedles under NIR irradiation for 210 s.

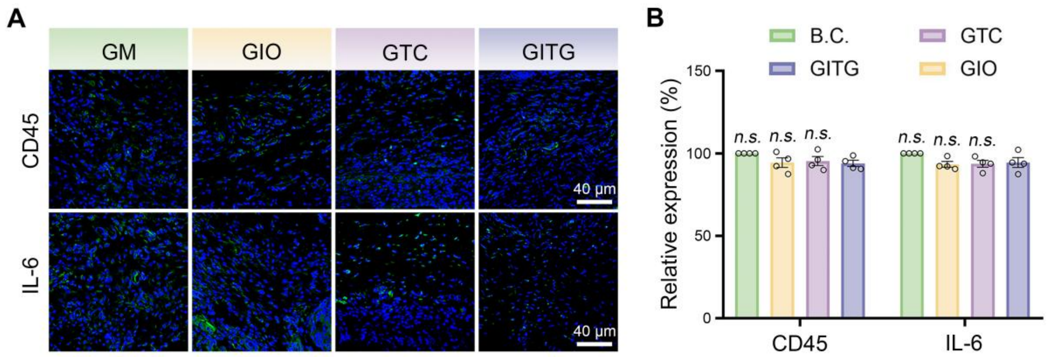

Figure S6. Evaluations of inflammation stage. (A) IF staining images of neo-capsule tissue. The expression of CD45 and IL-6 proteins was detected. Scale bar: 40 μm; (B) Quantitative results of IF staining images (n = 4). Values are expressed as the mean ± SD. Compared to GITG group, *n.s.* indicates no significance.

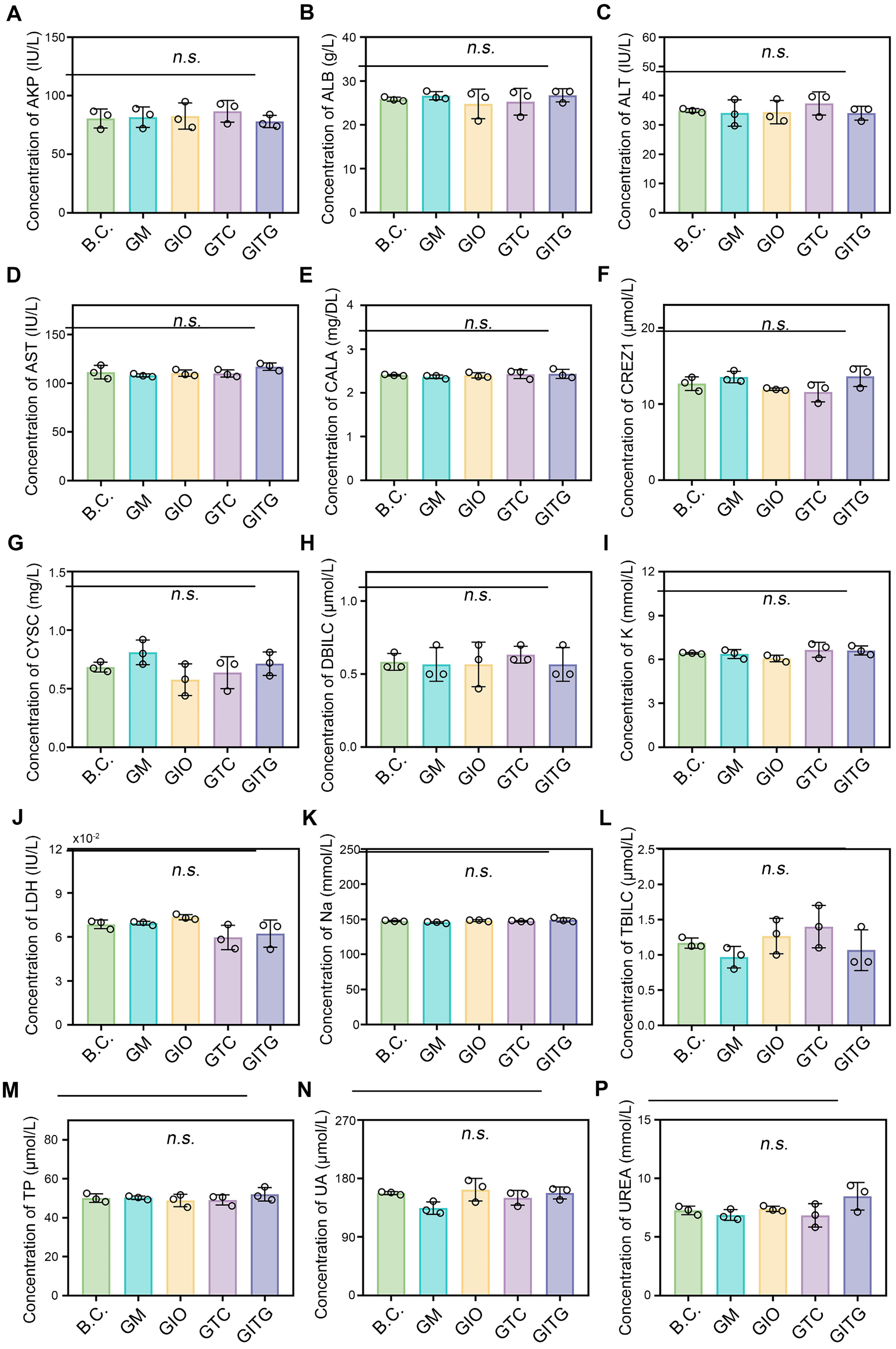

Figure S7. Blood biochemical tests (n = 3). Values are expressed as the mean ± SD. *n.s.* indicates no significance.

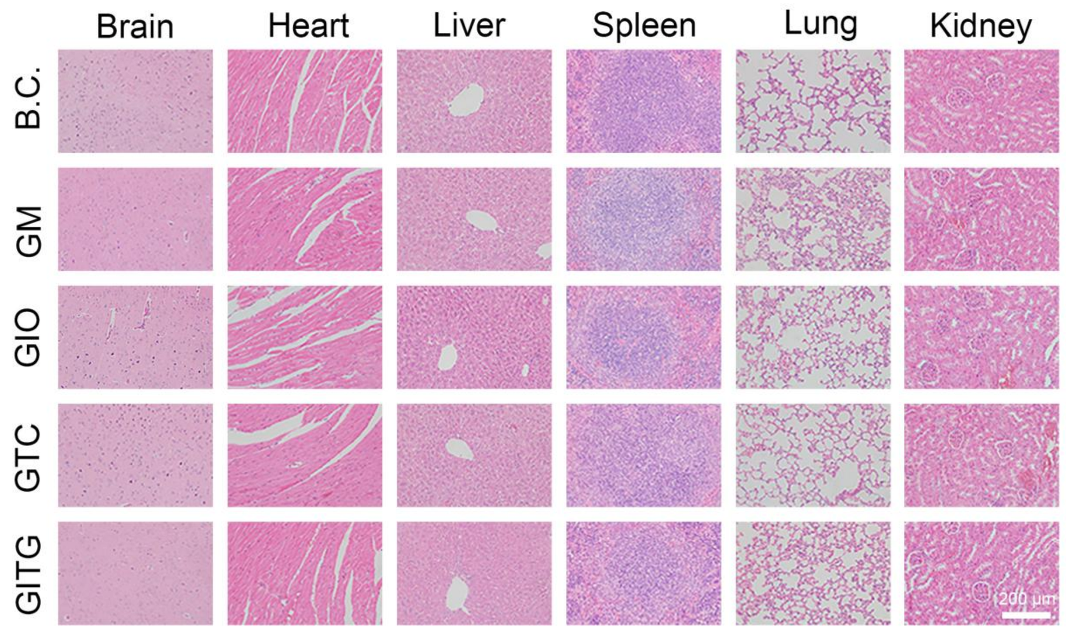

Figure S8. H&E staining images of the organs resected from the SD rats. Scale bar: 200 μm.

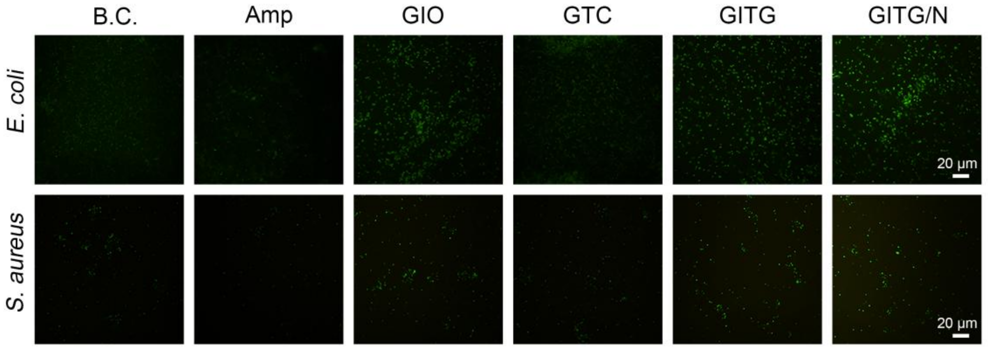

Figure S9. ROS staining images of different treatments. Scale bar: 20 μm.

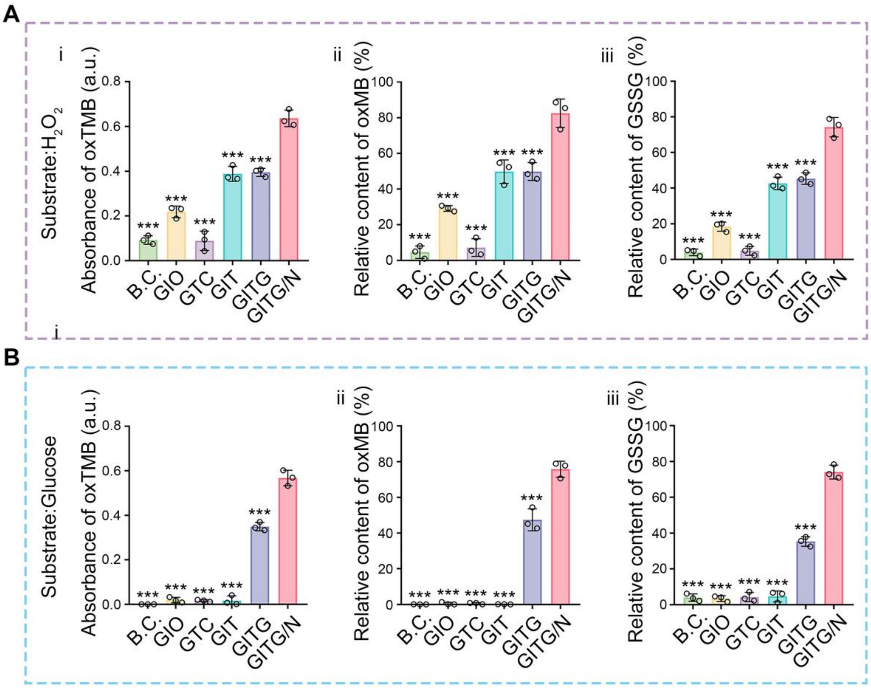

Figure S10. Pro-oxidative nanozyme-like activity. (A) Quantitative results of TMB assay, MB assay and DTNB assay using a substrate of H_2O_2 (n = 3); (B) Quantitative results of TMB assay, MB assay and DTNB assay using a substrate of glucose (n = 3). Values are expressed as the mean ± SD. Compared to GITG/N group, ***$P < 0.001$.

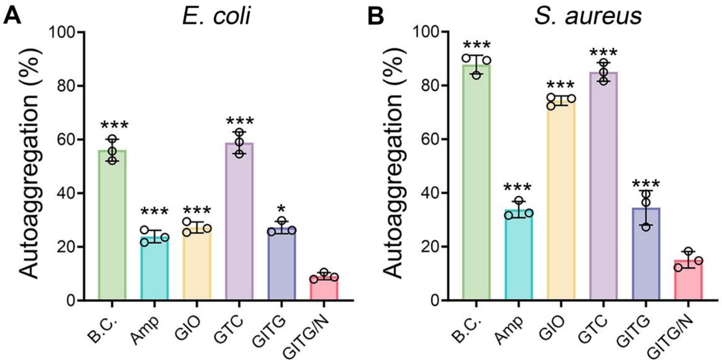

Figure S11. The bacterial autoaggregation of different treatments. (A) The autoaggregation of *E. coli.* (B) The autoaggregation of *S. aureus*. The values are expressed as the mean ± standard deviation (SD). Compared with the GITG/N group, *$P < 0.05$, and ***$P < 0.001$.

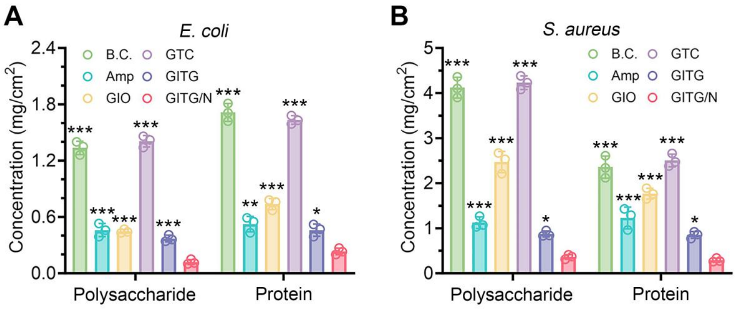

Figure S12.The polysaccharide and protein (EPS) concentration on biofilms of different Treatments. (A) The polysaccharide and protein concentration on biofilms concentration of *E. coli.* (B) The polysaccharide and protein concentration on biofilms of *S. aureus*. The values are expressed as the mean ± standard deviation (SD). Compared with the GITG/N group, *$P < 0.05$, *$P< 0.01$ and ***$P < 0.001$.

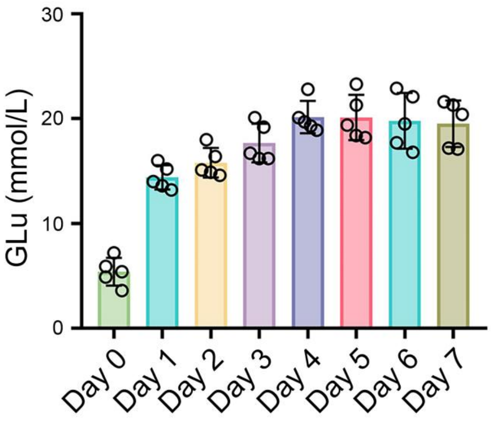

Figure S13. The content of blood glucose after STZ administration (n = 5).

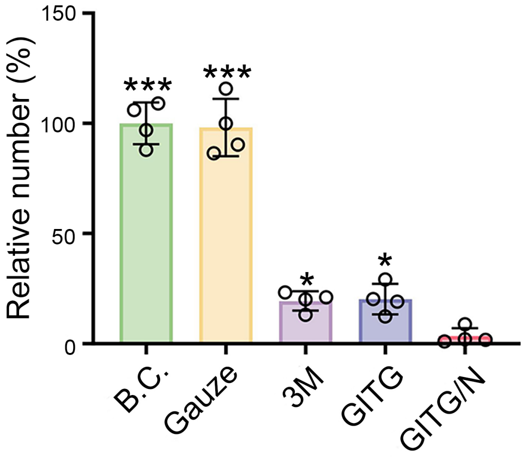

Figure S14. The number of bacterial clones (n = 4). Values are expressed as the mean ± SD. Compared to GITG/N group, ****P* < 0.001, **P* < 0.05.

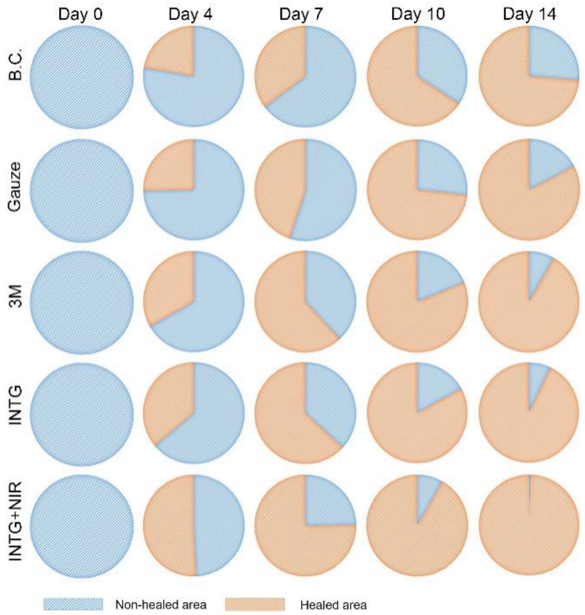

Figure S15. The wound healing rate of each wound image corresponding to Figure 6c.

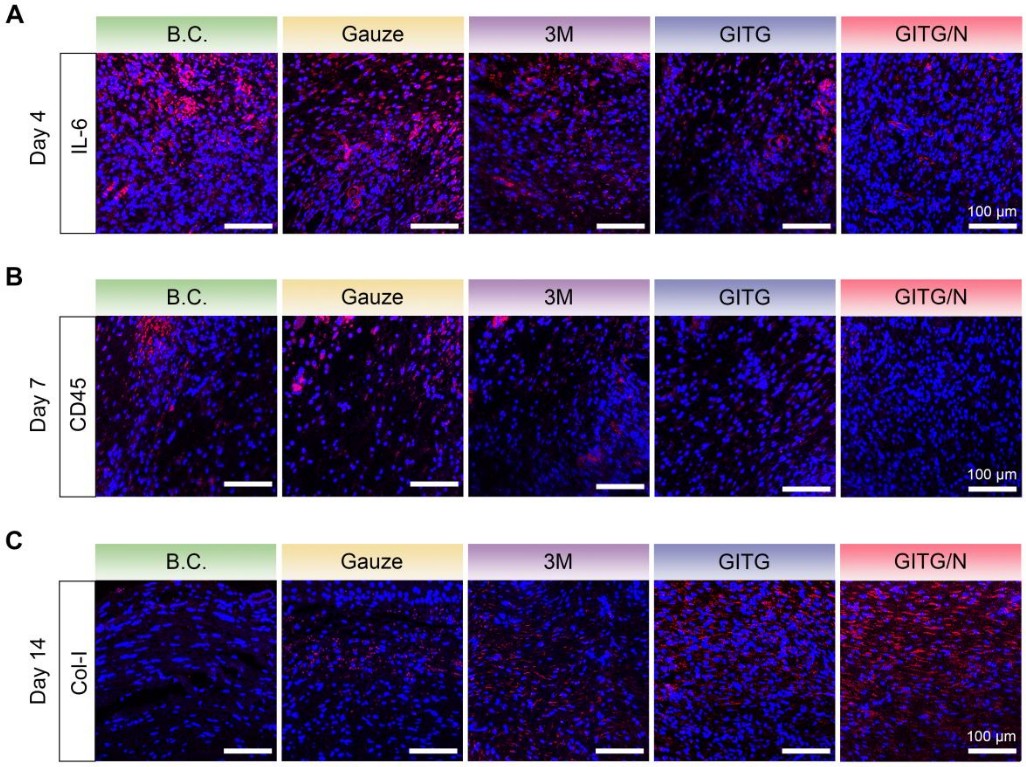

Figure S16. IF images of neo-skin tissues. (A) IL-6 on day 4. Scale bar: 100 μm; (B) CD45 on day 7. Scale bar: 100 μm; (C) Col-I on day 14. Scale bar: 100 μm.
